# Female‐biased astrocytic priming shapes early locus coeruleus vulnerability in an Aβ oligomer milieu

**DOI:** 10.1002/alz.71168

**Published:** 2026-02-06

**Authors:** Srishti Kushwaha, Rupsa Roy Choudhury, Priyanka Bhat, S. Senthil Kumaran, Smitha Karunakaran

**Affiliations:** ^1^ Centre for Brain Research Indian Institute of Science Bangalore India; ^2^ Manipal Academy of Higher Education Manipal India; ^3^ Department of NMR All India Institute of Medical Sciences New Delhi India

**Keywords:** alpha 2A‐adrenergic receptors, amyloid beta oligomers, astrocytic activation, environmental enrichment, locus coeruleus, noradrenergic signaling, norepinephrine transporter, preclinical Alzheimer's disease, proton magnetic resonance spectroscopy, sex differences

## Abstract

**INTRODUCTION:**

The locus coeruleus (LC) is an early site of Alzheimer's disease (AD) pathology, yet the role of brainstem astrocytes in early, sex‐dependent vulnerability remains unclear.

**METHODS:**

In 2‐ to 3‐month‐old APP/PS1 mice, we combined in vivo proton magnetic resonance spectroscopy (MRS) of the brainstem with region‐resolved molecular analyses, including quantitative real‐time polymerase chain reaction, amyloid beta 42 (Aβ42) oligomers enzyme‐linked immunosorbent assay, lactate assay, immunohistochemistry, immunoblotting, astrocyte isolation, and 3D structural assessment. Environmental enrichment (EE) served as a non‐pharmacologic intervention.

**RESULTS:**

Females exhibited higher brainstem Aβ42 oligomers and an astrocyte‐weighted MRS profile. Pontine glial fibrillary acidic protein (GFAP), complement component 3, and nuclear factor kappa‐light‐chain‐enhancer of activated B cells were selectively upregulated without pan‐reactive astrocytic and microglial markers. LC‐restricted GFAP elevation occurred without changes in astrocyte counts or morphology, indicating a “primed” state. Females also showed higher lactate levels, increased monocarboxylate transporter 2 expression, and elevations in selected oxidative phosphorylation‐associated transcripts, and reduced astrocytic alpha 2A‐adrenergic receptor expression. EE normalized noradrenergic and pontine astrocytic changes.

**DISCUSSION:**

Female‐biased, LC‐centric astrocytic priming emerges early in this amyloid‐driven model and is modifiable.

## Highlights

- Early astrocytic priming in locus coeruleus (LC) of APP/PS1 mice is stronger in females.
- Non‐reactive astrocytes show glial fibrillary acidic protein, complement component 3, nuclear factor kappa‐light‐chain‐enhancer of activated B cells upregulation with intact LC neurons.
- Females show higher glutamine, myo‐inositol, glutathione, and taurine levels.
- Noradrenergic tuning adapts in males via NET/α2A receptors but is impaired in females.
- Environmental enrichment restores LC astrocytic state, metabolic profile, and noradrenergic regulation.

## 1 BACKGROUND

Alzheimer's disease (AD) is the leading cause of dementia worldwide, with the fastest growth in low‐ and middle‐income countries where roughly two thirds of affected individuals now live.^1^ Women face higher risk and faster progression once symptoms appear.^2^, ^3^ AD begins decades before diagnosis–often 20 to 30 years earlier^4^–yet the earliest neural substrates and the biological bases of sex differences remain unclear. Evidence points to the brainstem, especially the locus coeruleus (LC), as an initial site of disease: tau pathology emerges there years before the entorhinal cortex, brainstem atrophy predicts dementia, and early brainstem changes link to prodromal symptoms like sleep disturbances and depression.^5^, ^6^, ^7^, ^8^ These findings suggest brainstem pathology arises in asymptomatic individuals and may be modulated by sex. Astrocytes, like neurons, are heterogeneous and undergo state transitions that influence resilience or vulnerability. We propose that maladaptive astrocyte transitions in the LC contribute to early, sex‐linked susceptibility.

Astrocytes orchestrate synaptic support, metabolic coupling, and neuroimmune signaling. Glial fibrillary acidic protein (GFAP) is a canonical marker of astrocytic cytoskeletal remodeling. Plasma GFAP levels rise across the AD continuum, although the relative contributions of central versus peripheral sources remain debated,^9^, ^10^ and the relationship between early non‐reactive astrocytic states and circulating GFAP is still unclear. Astrocytes can promote pathology through several routes: increased α2‐Na^+^/K^+^‐ATPase expression drives neuroinflammation and tau accumulation;^11^ oligomeric amyloid beta (Aβ) induces astrocytic complement component 3 (C3), signaling via neuronal C3aR to remodel neuronal dendrites; and dysregulated glutamate handling promotes excitotoxicity.^12^, ^13^, ^14^ These pathways support an astrocyte‐first contribution to early AD.

The LC–norepinephrine (NE) system intersects these mechanisms. LC degeneration is an early feature of AD, and noradrenergic loss correlates with cognitive decline and neuropsychiatric symptoms.^15^, ^16^ Noradrenergic tone exerts stage‐dependent effects: in aging and preclinical AD, higher central NE associates with better memory,^17^, ^18^ whereas in symptomatic stages elevated NE tracks with neuropsychiatric burden.^19^, ^20^ LC–NE also programs astrocyte biology, inducing transcriptional changes, promoting glycogen synthesis, and enabling lactate shuttling during learning.^21^ These functions position LC astrocytes as regulators of both neuromodulatory tone and local metabolic support.

Sex adds a critical layer to LC biology. Although AD shows clear sex differences in incidence, progression, and biomarker trajectories,^2^, ^3^, ^22^ anatomical sex differences in the human LC remain uncertain due to limited stereological power.^23^, ^24^ Imaging measures such as fluorodeoxyglucose positron emission tomography^25^ and high‐field LC‐magnetic resonance imaging^26^ report subtle sex‐linked signal differences. Rodent studies show sex‐dependent LC cell numbers,^27^, ^28^ though these trends may not generalize to humans. Preserved LC integrity—even with high Aβ burden—associates with better cognition.^25^, ^29^ While gonadal hormones influence neural and glial states, large trials like the Women's Health Initiative found increased dementia risk with hormone therapy, suggesting additional factors drive female‐biased risk.^30^, ^31^ Sex‐specific responses in anti‐Aβ trials (e.g., aducanumab EMERGE; lecanemab) further underscore biological heterogeneity.^32^

**RESEARCH IN CONTEXT**

**Systematic review**: We reviewed early Alzheimer's disease (AD) literature on brainstem vulnerability, locus coeruleus (LC) pathology, and sex‐specific glial responses. While studies show early tau buildup in the LC, sex‐biased plasma glial fibrillary acidic protein elevations, and glial roles in AD progression, astrocyte involvement in early LC‐related dysfunction remains unclear.

**Interpretation**: Our findings identify LC‐centered, astrocytic alterations in young APP/PS1 mice, more prominent in females, that emerges without neuronal loss or microgliosis. This state is distinct from classical reactive gliosis, aligning with a primed astrocytic state within the limits of this amyloid‐centric model. Environmental enrichment mitigated these early astrocytic and lactate–oxidative phosphorylation‐related shifts, indicating that such early alterations may be modifiable.

**Future directions**: Priorities include identifying the drivers of sex‐divergent LC astrocytic priming, testing whether similar early changes are detectable in human at‐risk cohorts, and determining if interventions that preserve LC astrocyte–neuron support pathways can extend cognitive resilience in more advanced or tau‐inclusive models.

These gaps support a brainstem‐first, astrocyte‐focused view of preclinical AD with explicit attention to sex. If the LC is among the earliest affected sites, astrocytic transitions before classical reactivity could dictate neuronal vulnerability and noradrenergic balance. Detecting GFAP‐upregulated but non‐reactive astrocytes at this stage may offer an early biomarker and therapeutic window.

We combined in vivo brainstem proton magnetic resonance spectroscopy (^1^H‐MRS) with region‐specific molecular profiling in 2‐ to ‐3‐month‐old APPswe/PS1dE9 mice—a stage with oligomeric Aβ but without tau pathology or neurodegeneration^33^—to: (1) map sex‐specific astrocytic and microglial signatures in the pons and LC, (2) quantify LC–NE regulation through the norepinephrine transporter (NET) and alpha 2A‐adrenergic receptors (α2ARs), and (3) test environmental enrichment (EE) as a non‐pharmacologic intervention. Results reveal LC‐centered astrocytic alterations at this early, amyloid‐driven stage, including a female‐biased, astrocytic‐inflammatory shift despite intact LC neurons. We also identify sex‐divergent neuron–astrocyte noradrenergic adaptations and show that EE normalizes multiple LC‐associated astrocytic and neuromodulatory changes, indicating these early alterations are modifiable.

## 2 METHODS

### 2.1 Experimental animals

Double transgenic mice B6C3‐Tg (APPSwe/PSEN1dE9) 85Dbo/J harboring mutations–APP: KM670/671NL (Swedish) and PSEN1: delta E9 were originally procured from The Jackson Laboratory (MMRRC Strain #034832‐JAX; RRID:MMRRC_034832‐JAX). The breeding of wild‐type (WT) and APPSwe/PSEN1dE9 (APP/PS1) mice, generation, and maintenance of colonies was done in the Central Animal Facility (CAF), Indian Institute of Science, Bangalore, India. They were housed under conventional laboratory conditions (12 hour dark and 12 hour light cycle, constant temperature, and humidity) with recommended diet and water ad libitum. 2‐ to 3‐month‐old male and female WT and APP/PS1 mice were used for the experiments. Female mice were used without estrous cycle staging and variability in the data due to random cycle stages is expected. All experimental procedures involved in the methodology were approved by the institutional animal ethics committee (IAEC; approval number: CAF/Ethics/919/2022) of the Indian Institute of Science and are in accordance with the guide for the care and use of laboratory animals. All efforts were made to reduce suffering of mice and the number of mice used for our required experiments.

No formal power calculation for sample size determination was performed a priori; group sizes reflect standard practice for comparable histological and molecular studies in transgenic mice. No samples were excluded from either the experiments or the analysis, unless otherwise mentioned in the figure legends. Sample sizes (*n* per group) are provided in each figure legend.

All the animal experiments were designed and followed in compliance with the Animal Research: Reporting In Vivo Experiments guidelines and applying double‐blinded analysis when possible. In experiments involving WT and APP/PS1 mice, the animals were assigned randomly to the respective groups based on the genotype.

### 2.2 Magnetic resonance imaging and spectroscopy

Magnetic resonance imaging (MRI) scans were acquired on a 7 Tesla MRI scanner (Biospec 70/20 US, M/s Bruker Biospin GmBH), using a volume coil (Transmit/Receive, inner diameter 40 mm). Mice were cleaned with sterillium to remove any residual fecal matter and husk particles before placement. Prior to the experiment, mice were initially anesthetized with isoflurane/O_2_ mixture inside a small chamber custom made specially for anesthetizing small animals. A Forane 100 vaporizer by Surgivet (vaporizer anesthetic system for veterinary purposes) was used to mix O_2_ and air with isoflurane. The anesthetic support was administered at a flow rate of 2 mL/minute for induction. The mice were then placed in the scanner bore in headfirst position. A custom‐made tether for mice was used to restrict the head motion and maintain optimal positioning during scanning protocol. During the scans, respiration was monitored using a physiological monitoring and gating assembly (Model 1030, SA Instruments Inc.). The maintenance dose of anesthesia was adjusted at an optimal flow rate of 0.5 to 1 mL/minute for maintenance during the scanning to achieve a respiratory rate of 30 to 40 breaths per minute. After the planner/localizer sequences, structural T1 sequences were acquired to assess optimal positioning of the animal in the field of view (FOV). T1 scans were thereafter acquired using a T1 rapid acquisition and relaxation enhancement (RARE) sequence in all three planes (coronal, axial, and sagittal) with an echo time (TE) = 8 ms, repetition time (TR) = 1500 ms, number of signal average (NSA) = 2, RARE factor = 4. The ^1^H single voxel (voxel size = 3 x 3 x 3 mm^3^) spectroscopy was planned on the T1 scans in the brainstem region to accommodate LC (Figure S1 in supporting information), with TE = 16.6 ms, TR = 2500 ms; NSA = 256, water suppression = CHESS (chemical shift selective; at bandwidth of 200 Hz).

#### 2.2.1 Data analysis of spectroscopy

MRS data were analyzed using LCModel, with an appropriate basis set of 7T provided with the software. The raw data were selected, fitted, and the spectra were processed for each MRS data (Figure S1). Metabolites were thresholded based on Cramér–Rao lower bounds (CRLB). Metabolites with CRLB > 20% were excluded from the analysis. The metabolite concentration ratios thus obtained were assessed for significance at group level. Inferences were arrived at, after analyzing using GraphPad Prism using two‐way analysis of variance (ANOVA) followed by Tukey post hoc analysis.

### 2.3 Western blotting

Mice were sacrificed by cervical dislocation, and pons and midbrain tissues were rapidly dissected, frozen in liquid nitrogen, and stored at −80°C. Tissues were homogenized using a Potter–Elvehjem homogenizer in a 1:9 volume of ice‐cold, freshly prepared brain homogenization buffer (NaCl, KCl, MgSO_4_, CaCl_2_, KH_2_PO_4_, glucose, and dithiothreitol [DTT]; pH 7.4) supplemented with protease inhibitor cocktail (10 µL/mL) and phosphatase inhibitors (sodium fluoride, sodium orthovanadate, and β‐glycerophosphate) added immediately before homogenization. Homogenates were centrifuged at 1000 × g for 10 minutes at 4°C, and protein concentration in the post‐nuclear supernatant was determined using the Pierce™ BCA Protein Assay Kit (Thermo Fisher Scientific: 23227). Each sample was mixed with 5x sodium dodecyl sulfate (SDS) loading buffer (Tris‐HCl, SDS, bromophenol blue, glycerol, DTT; pH 6.8), heated at 95°C for 5 minutes, aliquoted, and stored at −80°C. Frozen aliquots were thawed on ice for 15 minutes, heated at 70°C for 5 minutes, and equal protein amounts were resolved on 10% SDS‐polyacrylamide gel electrophoresis, followed by transfer onto polyvinylidene fluoride membranes (wet transfer, Tris‐glycine buffer with 20% methanol, 80 V, 90 minutes). Membranes were blocked in 5% skim milk in 1x Tris‐buffered saline (TBS) with 0.1% Tween‐20 (TBST) for 1 hour at room temperature, then incubated overnight at 4°C with primary antibodies diluted in 3% bovine serum albumin (BSA) in TBST: rabbit monoclonal anti‐dopamine‐β‐hydroxylase (DBH, 1:1000, abcam, ab209487), sheep polyclonal anti‐tyrosine hydroxylase (TH, 1:1000, Merck, AB1524), and rabbit monoclonal anti‐β‐tubulin (1:1000; Abclonal, A12289). Blots were washed three times with TBST, incubated with horseradish peroxidase–conjugated secondary antibody (1:4000) for 1 hour at room temperature, washed again three times, and developed using enhanced chemiluminescence. Bands were visualized with a ChemiDoc system and quantified using Image Lab software (Bio‐Rad), and data were analyzed with GraphPad Prism.

### 2.4 Aβ42 oligomer estimation

Brainstems (≈ 100 mg tissue per animal) were dissected and homogenized on ice using a Dounce homogenizer in extraction buffer containing 5 M guanidine hydrochloride and 50 mM Tris‐HCl (pH 8.0). Homogenates were incubated at room temperature for 3 to 4 hours with constant mixing to ensure complete solubilization of Aβ species. Aβ42 oligomer concentrations were quantified using the SensoLyte Anti‐Mouse/Rat β‐Amyloid (1‐42) Quantitative, Colorimetric Kit (AnaSpec, AS‐5554), following the manufacturer's instructions.

### 2.5 Lactate assay

L‐lactate levels were measured using the colorimetric L‐Lactate Assay Kit (abcam, ab65331) according to the manufacturer's instructions. Pons tissue was dissected, deproteinized on ice with 4 M perchloric acid (3:1) using a Dounce homogenizer and neutralized with 2 M potassium hydroxide. The resulting lysate was centrifuged, and the supernatant was used directly for L‐lactate quantification. Optical density was measured at 450 nm.

### 2.6 RNA isolation and quantitative real‐time polymerase chain reaction

Mice were euthanized by cervical dislocation, and the pons and midbrain were rapidly dissected on ice and flash‐frozen in liquid nitrogen. Tissue was homogenized using a Potter–Elvehjem homogenizer, and total RNA was extracted using the QIAgen RNeasy Plus Universal Mini Kit (Catalog No. 73404). RNA integrity was verified by electrophoresis on a 1% agarose gel in 1× TBE buffer. One µg of RNA from each sample was reverse‐transcribed into cDNA using the High‐Capacity cDNA Reverse Transcription Kit (Thermo Scientific, 4368814). Quantitative polymerase chain reaction (qPCR) was carried out with PowerUp SYBR Green Master Mix (Applied Biosystems, A‐25742) on a QuantStudio 7 Pro Real‐Time PCR system (Applied Biosystems). Relative gene expression was determined using the ΔΔ*CT* method. The primers used in this study are listed in Table S1 in supporting information.

### 2.7 Astrocyte isolation

Pons tissue from eight mice per group was dissected and pooled for astrocyte isolation. Tissue dissociation and enzymatic digestion were performed using the Adult Brain Dissociation Kit, mouse and rat (Miltenyi Biotec, 130‐107‐677) with the gentleMACS Dissociator with heaters (Miltenyi Biotec, 130‐096‐427) running program 37C_ABDK_02 for 30 minutes. Each sample was processed in a separate gentleMACS C tube (Miltenyi Biotec, 130‐093‐237) according to the manufacturer's protocol. The resulting cell suspension was filtered through MACS Smart Strainers (70 µm; Miltenyi Biotec, 130‐110‐916) to remove clumps. Myelin and debris were removed using the debris removal solution and multiple centrifugation steps as per the manufacturer's instructions. The cell pellet was resuspended in phosphate‐buffered saline (PBS)/BSA 0.5% without Ca^2^^+^/Mg^2^^+^ (PB buffer) by gentle pipetting and used for magnetic labeling and astrocyte separation.

All Magnetic‐Activated Cell Sorting (MACS) steps were carried out at 4°C. Cells were incubated with FcR blocking reagent (Miltenyi Biotec, 130‐092‐575) at a 1:9 dilution for 10 minutes to prevent non‐specific antibody binding, followed by incubation with Anti‐GLAST or Astrocyte Cell Surface Antigen‐1 (ACSA‐1)‐Biotin (Miltenyi Biotec, 130‐095‐826) for 10 minutes. After washing with PB buffer, cells were pelleted at 300 × g for 10 minutes, resuspended in PB buffer, and incubated with Anti‐Biotin Microbeads (Miltenyi Biotec, 130‐095‐826) for 15 minutes. Cells were washed again in PB buffer, centrifuged at 300 × g for 10 minutes, and passed through LS columns (pre‐rinsed with PB buffer, Miltenyi Biotec, 130‐122‐729) in a MidiMACS separator (Miltenyi Biotec, 130‐042‐302) and further washed with PB buffer three times. The column was then removed from the separator, placed over a 15 mL tube, and the GLAST^+^ fraction (astrocyte‐enriched) was eluted by flushing with 5 mL PB buffer using a plunger. The flow‐through was collected after each wash and labelled as the astrocyte‐depleted fraction.

RNA was extracted from both astrocyte‐enriched (Ast^+ve^) and astrocyte‐depleted (Ast^‐ve^) fractions of pons tissue using the PicoPure RNA Isolation Kit (Thermo Scientific, KIT0204) according to the manufacturer's protocol. qPCR was performed as described above. qPCR products were separated on a 1% agarose gel for the microglial marker CD68, the housekeeping gene 18S rRNA, and the astrocytic marker GFAP in Ast^+ve^ and Ast^−ve^ fractions. Band intensities were quantified, normalized to 18S rRNA, and presented as relative expression levels (Figure S2A–C in supporting information). Visualization was performed using a ChemiDoc system, and quantification was carried out with ImageLab software (Bio‐Rad).

To optimize and validate the ACSA‐1–based magnetic enrichment procedure, an initial pilot flow‐cytometry analysis was performed using dissociated dorsal hippocampal tissue. This tissue was used solely for methodological standardization because it yields higher cell numbers and provides a stable preparation for testing antibody performance. Single‐cell suspensions and the corresponding ACSA‐1–enriched fraction obtained after LS‐column separation were stained with ACSA‐1–PE antibody (Miltenyi Biotec, 130‐118‐483) and analyzed together using standard forward scatter/side scatter gating to exclude debris. In this optimization dataset, the unsorted suspension contained ≈ 24% ACSA‐1^+ve^ cells, whereas the enriched fraction contained ≈ 58% ACSA‐1^+ve^ cells, confirming that the positive‐selection protocol effectively increased astrocyte representation (Figure S2D, E). A depletion fraction was not examined in this pilot. These data were used to validate the isolation workflow; purity of the experimental pontine isolates used for all molecular analyses was assessed independently by qPCR (GFAP enrichment relative to CD68), as described above.

### 2.8 Early‐life (post‐weaning) environmental enrichment

Early‐life EE was performed following established protocols.^34^ After weaning (postnatal day 21, P21), male and female WT and APP/PS1 mice aged 26 days were housed in enriched cages (19.5 × 14 × 7 inches) containing multiple toys, including running wheels, tunnels, and various objects of different shapes and sizes, with ad libitum access to food and water. Mice remained in this environment for 21 days, with objects replaced or repositioned every third day. At P47, mice were transferred to standard group housing containing only bedding, food, and water until P60. Control mice were maintained in standard home cages for the entire duration of the experiment (Figure S3 in supporting information).

### 2.9 Tissue processing and immunohistochemistry

Mice were anesthetized with isoflurane and perfused transcardially with 1x PBS (pH 7.4), followed by 4% paraformaldehyde (PFA). Brains were removed, post‐fixed overnight in 4% PFA at 4°C, and cryoprotected in 30% sucrose at 4°C before cryosectioning. Coronal sections (50 µm) were cut using a cryostat (Leica, Germany), collected serially, stored in 1x PBS containing 0.02% sodium azide in 48‐well plates, and maintained at 4°C. Two sections per genotype were selected for the pons (Bregma: −5.52 and −5.68 mm) and midbrain (Bregma: −3.08 and −3.16 mm). Sections were mounted on charged slides, fixed at 37°C for 2.5 hours, washed in 1x PBS (5×5 minutes), and subjected to heat‐induced antigen retrieval in sodium citrate buffer (pH 6.0) using a pressure cooker when required. After retrieval, sections were washed (1x PBS, 5×5 minutes) and incubated for 1 hour in blocking serum (10% normal donkey serum in 1x PBS with 0.3% Triton X‐100) at room temperature in a humidified chamber. Sections were incubated overnight at 4°C with primary antibodies: mouse anti‐GFAP (1:300, Cell Signalling Technology, 3670), rabbit anti‐Iba1 (1:250, Cell Signalling Technology, 17198), rabbit anti‐Sox9 (1:500, Merck, AB5535), sheep anti‐TH (1:1000, Merck, AB1542), mouse anti‐NET (1:500, Abcam, ab211463), and rabbit anti‐α2AR (1:200, Abcam, ab85570). After primary incubation, sections were washed in PBST (PBS + 0.1% Triton X‐100, 3×15 minutes) and incubated for 2 hours at room temperature in the dark with secondary antibodies: Alexa Fluor™ 488 donkey anti‐mouse immunoglobulin G (IgG, 1:250, Invitrogen, A‐21202), Alexa Fluor™ 568 donkey anti‐rabbit IgG (1:250, Invitrogen, A‐10042), and Alexa Fluor™ 647 donkey anti‐sheep IgG (1:250, Invitrogen, A‐21448). Sections were washed in 1x PBST (3×5 minutes), rinsed in 1x PBS (1×5 minutes), and mounted in Fluoromount‐G™ with DAPI (00‐4959‐52). Imaging was performed on an Olympus Fluoview FV3000 Laser Scanning confocal microscope. Antibody specificity was supported by the use of commercially validated antibodies with established reactivity in mouse central nervous system tissue. Sections stained with normal IgG were used as negative controls for each staining protocol, which produced no detectable signal under identical imaging and acquisition parameters.

### 2.10 Image analysis

#### 2.10.1 LC volume estimation

Every alternate section spanning the rostro‐caudal extent of the LC (Bregma −5.32 to −5.80 mm) was TH‐stained and imaged at 20X. The Cavalieri principle was used to obtain an unbiased stereological estimation of the LC volume, as *V*  =  ∑A x T, where *∑A* is the sum of the areas measured in all LC sections and *T* is the distance between sections.^27^ The LC soma was manually outlined for area measurements in each section using the freehand tool on ImageJ (Fiji; https://imagej.nih.gov/ij/). The parallel LC images spaced 50 µm apart were stacked and analyzed. The resulting volume was expressed in cubic millimeters.

#### 2.10.2 Quantification of LC soma and TH fluorescence intensity

LC soma and TH fluorescence intensity were quantified from two sections per animal within Bregma −5.48 to −5.72 mm. Images were acquired at 10X, with *z* stacks (2.0 µm step size), standardized exposure times, and collapsed into maximum intensity projections (MIPs). Background subtraction (50‐pixel rolling ball) was applied, regions of interest (ROIs) were drawn around LC soma, and mean fluorescence intensity was measured in ImageJ.

#### 2.10.3 Quantification of GFAP and Iba1 fluorescence intensity

The mean fluorescence intensity of GFAP‐positive (GFAP^+ve^) astrocytes and Iba1‐positive (Iba^+ve^) microglia was quantified from multiple ROIs in the pons and the midbrain. Images were acquired at 10X magnification with *z* stacks of step size 2.0 µm and normalized to the same exposure time. The *z* stacks were collapsed to generate a 2D MIP image. ROIs were drawn per MIP image. The intensities were measured using ImageJ after subtracting the background fluorescence using a 50‐pixel rolling ball radius from the sections.

#### 2.10.4 Quantification of total astrocyte and microglial counts

The average counts of Sox9^+ve^ astrocytes and Iba1^+ve^ microglia were manually counted and normalized to their respective ROI volume in the LC region using the multi‐counter tool in ImageJ.

#### 2.10.5 Quantification of NET and total α2AR fluorescence intensity

NET and total α2AR intensities were quantified from multiple equal‐sized ROIs in the LC soma and dendritic regions. Images were acquired at 1 µm step size, at 40X magnification. ROIs were drawn per MIP image. The intensities were measured using ImageJ after subtracting the background fluorescence using a 50‐pixel rolling ball radius from the sections. Data were plotted separately for soma and dendrites and then averaged to yield a single value for the overall somatodendritic region.

#### 2.10.6 Quantification of α2AR density in astrocytes

Confocal images of LC soma and dendritic ROIs were acquired at 0.25 µm step size, at 60X magnification with oil immersion objective with 2X zoom, and were analyzed using MetaMorph software (Molecular Devices). Images saved as TIFF files were loaded on MetaMorph and calibrated in µm/pixel. MIP images were created using the Stack Arithmetic option. Average gray value, checked using the region statistics option, was used to threshold images. Binary masks of thresholded images were created and saved as 1‐bit files. Integrated Morphometry Analysis was done using MIP image of receptor as “source” and segmented using mask of 1‐bit binary image of astrocyte to measure receptor density in astrocytes. Object data and summary data of total area, average intensity, and total intensity were recorded. α2AR puncta density per GFAP^+ve^ astrocyte was reported as intensity/µm^2^. Data were plotted separately for soma astrocytes and dendritic astrocytes and then combined to yield the overall somatodendritic astrocyte α2AR density.

#### 2.10.7 3D reconstruction of LC astrocytes

Confocal images were acquired at 60X magnification at 0.25 µm step size with oil immersion objective. Twelve to nineteen astrocytes were analyzed per group using Imaris (Bitplane, 10.0.1). From the confocal image, an astrocyte of interest was cropped using the “crop 3D” function. The surface function was used to manually create a surface around the astrocyte of interest. After creation, the intensity of voxels outside the created surfaces was masked to zero using the “mask all” option. A masked channel containing the immunofluorescence of the astrocyte of interest was obtained. This masked channel was used to perform the 3D reconstruction of individual astrocytes using the “filament function.” The “tree autopath” algorithm was used to render the 3D filaments. The estimated starting point diameter for each image was obtained from the “slice mode” by clicking on two points across the diameter of the soma. The soma segmentation can be obtained as soma models or starting points. However, the soma model function does not work well for images with faint intensity around the soma, hence standard spheres set to the appropriate diameters were used. The estimated diameter for seed points was obtained in a similar manner from the “slice mode.” The automatic detection of seed points is rarely accurate and hence requires manual thresholding of the seed points. For further refinement, seed points were manually added or deleted by a left click of the mouse while holding down the shift button. The “remove seed points around starting points” function enabled mitigation of the creation of false hairlike projections around the edges of the soma with high intensity. Seed point classification and segment classification were performed to train the software for the predicted seed points and segments to keep and discard. Upon creation of 3D filament structure, the “edit” and “draw” tabs were used to edit falsely added and missing filaments. The “autopath” function under the “draw” tab was used to draw any missing filaments. The “edit” tab was used to delete false segments, join two segments to add missing beginning points, and to smooth the filaments created (Figure S4 in supporting information). All structural parameters including values of Sholl interactions were obtained from the “statistics” tab.

### 2.11 Statistical analysis

Statistical analyses were performed using Prism 10 (GraphPad Software Inc.). Normality of each dataset was evaluated using the Shapiro–Wilk test. When data were normally distributed and sample sizes permitted, parametric tests were applied; when normality was not satisfied or distributions suggested deviation from parametric assumptions, non‐parametric tests were used.

Two‐group comparisons were analyzed using two‐tailed unpaired Student *t* tests for normally distributed data, and Mann–Whitney *U* tests for non‐normal data or cases in which distributional assumptions could not be confidently verified given small sample sizes. Analyses involving two independent factors were performed using two‐way ANOVA, followed by Tukey post hoc tests. Data are presented as mean ± standard error of mean (SEM), and significance was set at *p* < 0.05. To aid interpretation under limited sample sizes, effect sizes are reported alongside *p* values: partial eta squared (*η* ^2^) for ANOVA models (with conventional thresholds: small 0.01, medium 0.06, large 0.14 for partial *η* ^2^).^35^

No a priori power calculations were performed; group sizes were constrained by feasibility of brainstem dissections and availability of sex‐balanced APP/PS1 cohorts. This limitation is acknowledged, and subtle effects may remain undetectable at the current *n*. Exact sample sizes (*n* per group) and statistical tests used for each analysis are provided in the corresponding figure legends and results text to ensure transparency and reproducibility.

## 3 RESULTS

### 3.1 Sex‐specific astrocyte‐related metabolic shifts in the brainstem of APP/PS1 mice revealed by in vivo ^1^H‐MRS

Metabolic profiling and amyloid–oligomer quantification in the brainstem of 2‐ to ‐3‐month‐old APP/PS1 mice revealed a sex‐dependent pattern of early changes within the constraints of this amyloid‐driven model. Brainstem Aβ42 oligomer levels showed a significant sex effect (Figure S5A in supporting information; two‐way ANOVA, *F*[1,13] = 5.223, *p *= 0.04, *ηp* ^2^ = 0.287), with female APP/PS1 mice exhibiting higher levels than male APP/PS1 mice (Tukey post hoc, *p *= 0.01). β‐site APP‐cleaving enzyme 1 (*BACE1*) levels were similarly elevated in female APP/PS1 mice (Figure S5B; Mann–Whitney *U* = 5, *p *= 0.041), indicating a sex‐biased amplification of amyloidogenic processing at this early stage.

**FIGURE 1:**
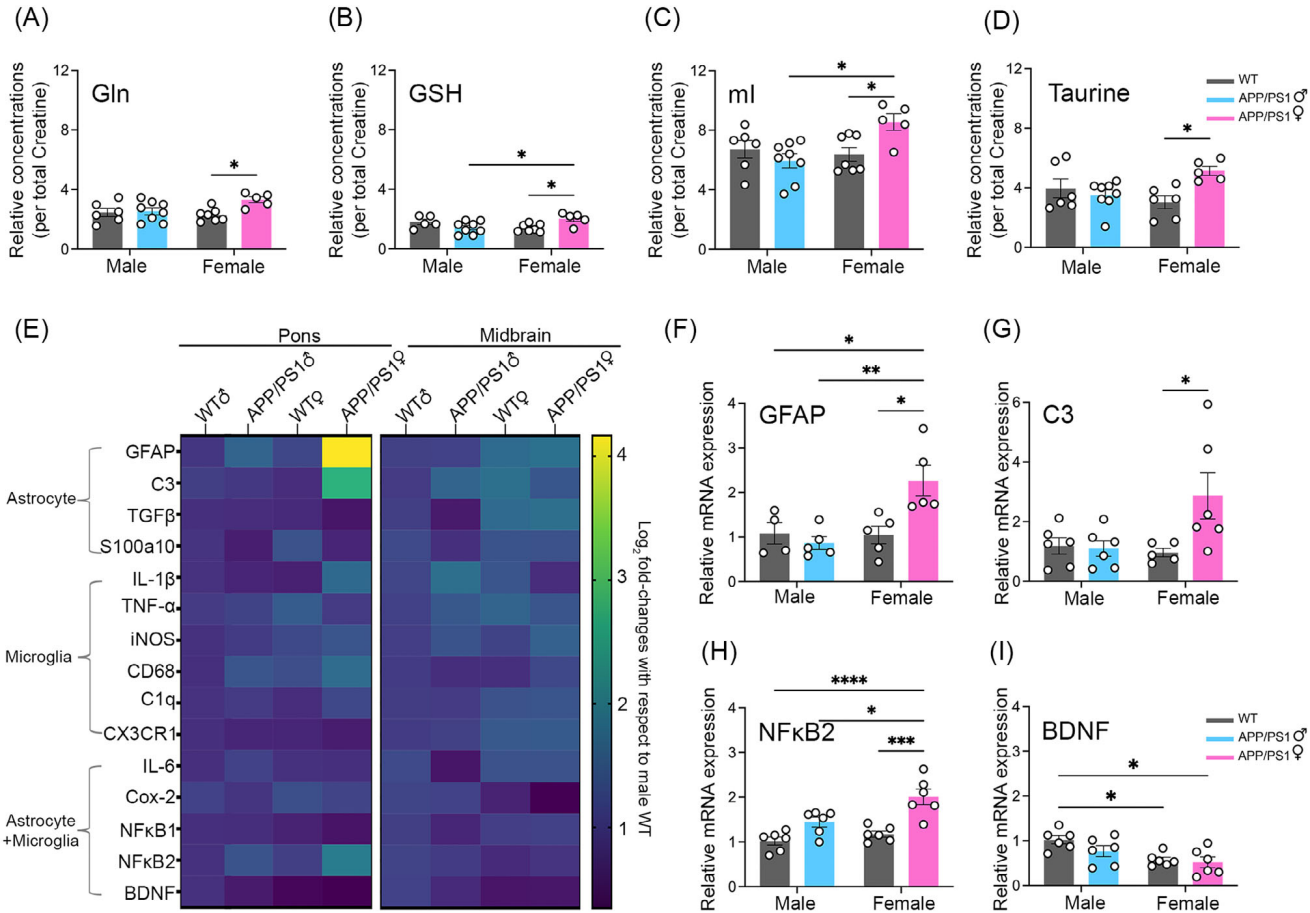
Astrocytic activation and altered metabolite profiles in the pons of 2‐ to 3‐month‐old female APP/PS1 mice. Brainstem metabolites were profiled using ^1^H‐MRS and reported as relative concentrations normalized to total creatine (tCr). Relative concentrations of (A) Gln, (B) GSH, (C) mI, and (D) taurine were significantly increased in female APP/PS1 mice compared to female WT (male WT, *n* = 6, male APP/PS1, *n* = 8, female WT, *n* = 7, female APP/PS1, *n* = 7). Data from two female APP/PS1 mice had to be excluded due to technical issue that interfered with MRS acquisition. Metabolite quantification was performed using LCModel, and metabolites with Cramér–Rao lower bounds (CRLB) > 20% were excluded from analysis (one male WT in GSH and one female WT in taurine). Data were analyzed using two‐way ANOVA with Tukey post hoc test; data shown as mean ± SEM; *p < 0.05. mRNA expression levels of proinflammatory and neuroprotective glial markers were quantified by qPCR in the pons and midbrain of 2‐ to 3‐month‐old male and female WT and APP/PS1 mice (*n* = 6 per group). E, Heatmap showing relative fold change in mRNA expression levels in the pons and midbrain normalized to male WT; values represent mean log_2_ fold‐changes calculated using ΔΔ*Ct* method; blue to yellow indicate increasing color gradient scale where dark blue indicates low expression and yellow indicates high expression, with color intensity reflecting the magnitude of expression. Relative mRNA expression level of (F) *GFAP*, (G) *C3*, (H) *NF‐κB2*, and (I) *BDNF* normalized to male WT. Female APP/PS1 mice exhibited significantly elevated *GFAP*, *C3*, and *NF‐κB2* expression in the pons, while *BDNF* expression was significantly reduced compared to male WT (*n* = 4–6; two‐way ANOVA with Tukey post hoc test; data shown as mean ± SEM; *p < 0.05, **p < 0.01, ***p < 0.001, ****p < 0.0001). qPCR was performed on *n* = 6 starting samples; samples with undetected signal were excluded from the *GFAP* and *C3* analyses. ^1^H‐MRS, proton magnetic resonance spectroscopy; ANOVA, analysis of variance; BDNF, brain‐derived neurotrophic factor; C3, complement component 3; GFAP, glial fibrillary acidic protein; Gln, glutamine; GSH, glutathione; IL, interleukin; iNOS, inducible nitric oxide synthase; mI, myo‐inositol; NF‐κB2, nuclear factor‐kappa‐B p100/p52 subunit; qPCR, quantitative polymerase chain reaction; SEM, standard error of mean; TgfGFβ, transforming growth factor beta; WT, wild type.

This oligomeric burden coincided with shifts in several astrocyte‐linked metabolites detected by in vivo ^1^H‐MRS (Table S2 in supporting information). Glutamine (Gln) levels showed a main effect of genotype (Figure 1A; *F*[1,22] = 5.534, *p *= 0.028, *ηp* ^2^ = 0.20). Tukey post hoc revealed higher Gln in female APP/PS1 than female WT (*p* = 0.033), representing a modest but directionally consistent increase (≈ 45%; Table S2) with moderate effect size, interpreted cautiously given sample size. Because astrocytes generate Gln through the glutamate–glutamine cycle, this pattern suggests early alteration in astrocyte‐associated metabolic regulation. Glutamate (Glu) and glutamate/glutamine (Glx) levels showed no significant main or interaction effects (Figure S5C,D).

Glutathione (GSH), for which astrocytes are a major source, showed a significant sex × genotype interaction (Figure 1B; *F*[1,22] = 10.85, *p *= 0.004, *ηp* ^2^ = 0.33). Female APP/PS1 mice exhibited higher GSH than male APP/PS1 and female WT (both *p *= 0.046), consistent with an early, sex‐specific increase in antioxidant capacity. Myo‐inositol (mI), often interpreted in MRS studies as reflecting glial or astrocytic content, was similarly elevated in female APP/PS1 mice (Figure 1C). mI showed a sex × genotype interaction (*F*[1,22] = 7.853, *p *= 0.01, *ηp* ^2^ = 0.263) and a main effect of sex (*p *= 0.043), with post hoc tests indicating higher mI in female APP/PS1 than male APP/PS1 (*p *= 0.011) or female WT (*p *= 0.047). Taurine, another astrocyte‐linked metabolite involved in osmoregulation, showed a significant sex × genotype interaction (Figure 1D; *F*[1,21] = 8.079, *p *= 0.01, *ηp* ^2^ = 0.278), driven by elevated taurine in female APP/PS1 mice (*p *= 0.024).

In contrast, neuronal and membrane‐related metabolites—total creatine (Cr+PCr), *N*‐acetylaspartate (NAA), *N*‐acetylaspartyl glutamate (NAA+NAAG), and glycerophosphocholine and phosphocholine (GPC+PCh)—showed no significant main or interaction effects (Figure S5E–H, Table S3 in supporting information), consistent with the absence of neurodegeneration in this model at this age.

Together, these findings suggest that early brainstem amyloid oligomer accumulation in females is accompanied by changes in metabolites enriched in astrocyte‐related metabolic pathways, including Gln, mI, GSH, and taurine. Because these metabolites are not exclusive to astrocytes, contributions from microglia or other cell types cannot be excluded. The results underscore a female‐biased pattern to early astrocyte‐linked metabolic and oxidative changes within this amyloid‐driven stage of the APP/PS1 model, driven by sex × genotype interactions rather than genotype alone (Table S2).

### 3.2 Selective GFAP‐only astrocytic activation with C3 and NF‐κB2 upregulation in the pons of female APP/PS1 mice

Based on the metabolite patterns observed with ^1^H‐MRS, we next examined transcriptional markers associated with astrocytes, microglia, and shared inflammatory pathways in the pons and midbrain (Figure 1E). To confirm regional specificity of our dissections, we quantified TH and dopamine β‐hydroxylase (DBH) by western blotting. As expected, TH expression was higher in the midbrain whereas DBH was higher in the pons (Figure S6A–C in supporting information; TH: *t*[10] = 4.232, *p *= 0.002; DBH: *t*[10] = 2.534, *p *= 0.03), supporting accurate anatomical isolation.

In the pons, *GFAP* expression showed a significant sex × genotype interaction (Figure 1F; *F*[1,15] = 8.460, *p *= 0.011, *ηp* ^2^ = 0.361). Female APP/PS1 mice exhibited higher *GFAP* mRNA than both male APP/PS1 mice (*p *= 0.004) and female WT (*p *= 0.013). To determine whether this reflected a broader reactive astrocyte profile, we assessed additional markers commonly associated with reactive astrocyte states (*vimentin*, *Serpina3n*, and *S100a10*). None showed significant changes (Figure S7A–C in supporting information; *p *> 0.05), indicating that the *GFAP* increase does not correspond to a full reactive transcriptional program and may instead represent an early or selective astrocytic shift.

We next evaluated astrocyte‐associated inflammatory markers (inducible nitric oxide synthase, transforming growth factor beta), neither of which differed across groups (Figure S8A,B in supporting information; *p *> 0.05). Microglia‐associated markers (*CD68, CX3CR1, C1q*), as well as cytokines typically upregulated during microglial activation (tumor necrosis factor α, interleukin [IL]‐1β), also showed no significant sex or genotype effects (Figure S8C‐G; *p *> 0.05). Similarly, transcripts expressed by both astrocytes and microglia (nuclear factor kappa‐light‐chain‐enhancer of activated B cells [*NF‐κB1*], *Cox‐2*, *IL‐6*) were unchanged (Figure S8H–J; *p *> 0.05). Together, these findings indicate no evidence of broad microglial activation or pan‐reactive astrocytosis in the pons at this early stage.

In contrast, *C3*—a complement component that can be expressed by astrocytes during inflammatory states—showed a significant sex × genotype interaction (Figure 1G; *F*[1,19] = 4.711, *p *= 0.043, *ηp* ^2^ = 0.199). Female APP/PS1 mice displayed higher *C3* expression than female WT (*p *= 0.045), with a trend toward elevation relative to male APP/PS1 mice (*p* = 0.052). *NF‐κB2* expression was also significantly elevated in the pons of female APP/PS1 mice, with independent contributions of sex and genotype (Figure 1H; sex: *F*[1,20] = 9.34, *p *= 0.006, *ηp* ^2^ = 0.318; genotype: *F*[1,20] = 28.58, *p *< 0.001, *ηp* ^2^ = 0.588).

As reported previously, brain‐derived neurotrophic factor (*BDNF*) expression exhibited sexual dimorphism in WT mice.^36^ Consistent with this, *BDNF* showed a significant main effect of sex (Figure 1I; *F*[1,20] = 11.63, *p *= 0.003, *ηp* ^2^ = 0.368), with lower levels in females; genotype and sex × genotype interaction were not significant. Thus, APP/PS1 status did not alter the underlying sex‐dependent pattern of *BDNF* expression.

No significant changes in astrocyte‐associated, microglia‐associated, or shared inflammatory markers were detected in the midbrain (Figure S9A–O in supporting information; *p *> 0.05). Overall, these data identify the pons of female APP/PS1 mice as a site of selective transcriptional upregulation of, *C3*, and *NFκB2* in the absence of microglial activation or pan‐reactive astrocyte signatures.

**FIGURE 2:**
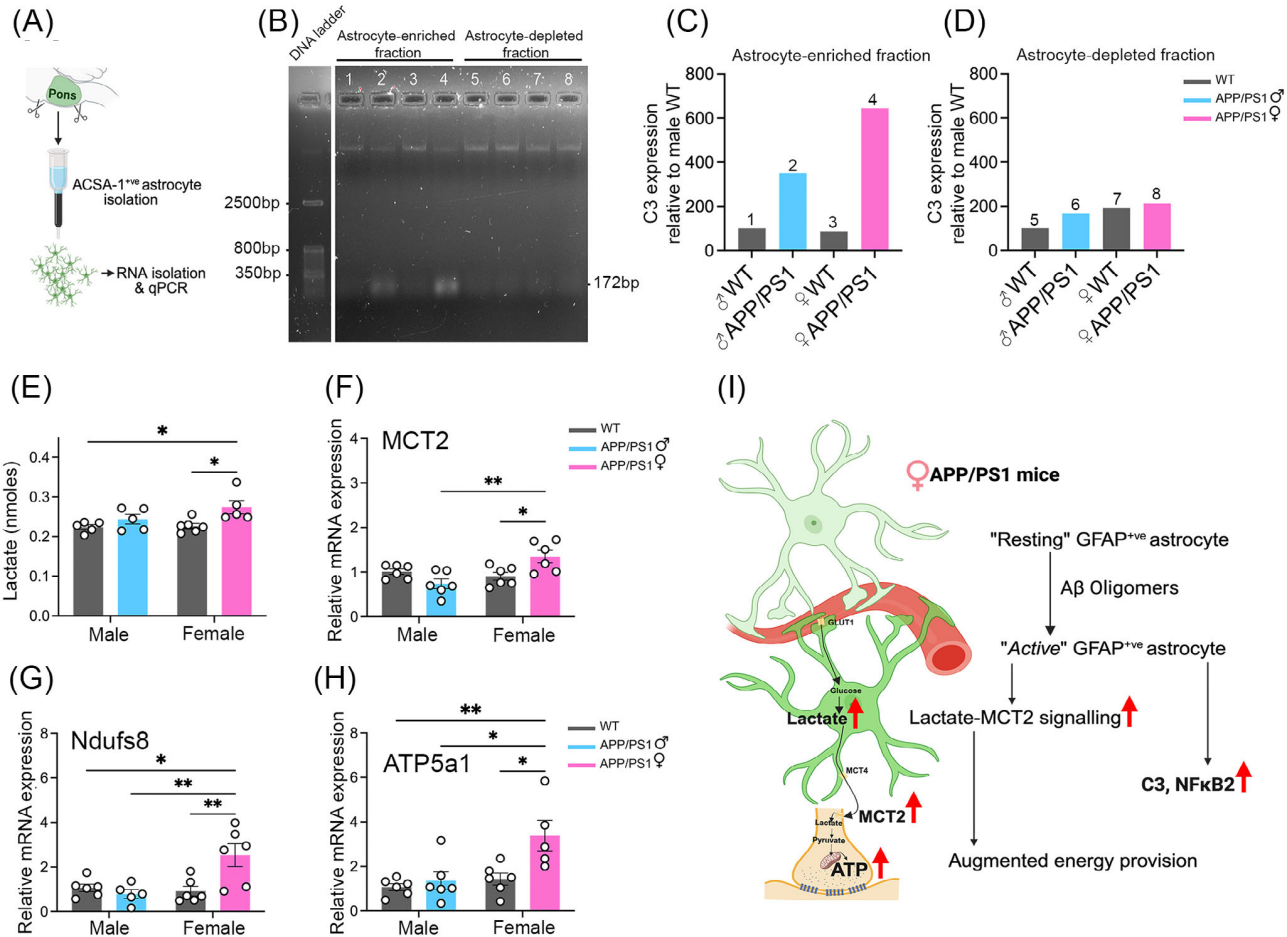
Astrocyte‐linked C3 elevation and lactate–MCT2–OXPHOS axis engagement in the pons of female APP/PS1 mice. Astrocyte‐enriched pons fraction shows increased *C3* expression in 2‐ to 3‐month‐old female APP/PS1 mice. A, Schematic illustrating MACS‐based isolation of pontine astrocytes. Schematic created using Biorender. B, Assessment of *C3* expression by qPCR followed by 1% agarose gel electrophoresis–gel image showing *C3* bands at 172 bp in astrocyte‐enriched fractions (lane 1–4) and astrocyte‐depleted fractions (lane 5–8). C, D, Bar graphs demonstrating elevated *C3* levels in the astrocyte‐enriched fraction of female APP/PS1 mice compared to other groups and to their corresponding negative fraction. Each bar represents pooled pons samples from 8 mice per group. Lactate–MCT2 axis and OXPHOS pathway engagement in female APP/PS1 mice. Female APP/PS1 mice showed higher (E) lactate levels in the pons measured by colorimetric assay and increased (F) *MCT2* expression assessed by qPCR (*n* = 6 per group; two‐way ANOVA with Tukey post hoc test; data shown as mean ± SEM; *p < 0.05, **p < 0.01). G, H, mRNA expression profiling of mitochondrial markers by qPCR in the pons. Female APP/PS1 mice showed significantly elevated expression of the mitochondrial complex I subunit *Ndufs8* and complex V subunit *Atp5a1*, indicating adaptive mitochondrial activation in the pons (*n* = 5‐6; two‐way ANOVA with Tukey post hoc test; data shown as mean ± SEM; *p < 0.05, **p < 0.01). I, Graphical summary. Elevated Aβ oligomers in female APP/PS1 mice shift astrocytes from a resting to an active GFAP^+ve^ state, with associated *C3* and *NF‐κB2* upregulation and engagement of lactate–OXPHOS axis, supporting increased energetic demands. Graphical summary created using Biorender. Aβ, amyloid beta; ACSA‐1, astrocyte cell surface antigen 1; ANOVA, analysis of variance; Atp5a1, ATP synthase F1 subunit α; C3, complement component 3; GLUT1, glucose transporter 1; MACS, magnetic‐activated cell sorting; MCT2, monocarboxylate transporter 2; Ndufs8, NADH dehydrogenase iron‐sulfur protein 8; NF‐κB2, nuclear factor‐kappa‐B p100/p52 subunit; OXPHOS, oxidative phosphorylation; qPCR, quantitative polymerase chain reaction; SEM, standard error of mean; WT, wild type.

To examine whether astrocytes might contribute to the elevated *C3* signal, we performed ACSA‐1–based MACS isolation of astrocyte‐enriched and astrocyte‐depleted fractions from pooled pons tissue (8 mice per group, *n* = 1). Enrichment quality was verified by higher *GFAP* and lower *CD68* expression in the enriched fraction relative to the depleted fraction (Figure S2A–C). Because each genotype pool yielded a single enriched and single depleted sample (*n* = 1 per group), these data are not compared statistically to infer group differences. Within this qualitative dataset, *C3* levels appeared higher in the astrocyte‐enriched fraction from female APP/PS1 mice (Figures 2A–D, and S10 in supporting information); however, we interpret this only as confirmation that the astrocyte enrichment procedure captures *C3*‐expressing cells, and we do not draw biological conclusions from these pooled samples. Interpretation of genotype effects relies exclusively on the bulk qPCR data.

### 3.3 Sex‐dependent pontine astrocytic lactate–oxidative phosphorylation coupling in female APP/PS1 mice

As both the MRS in the brainstem, and qPCR analyses in the pons, pointed to a selective astrocyte‐related shift in female APP/PS1 mice, we next examined whether these molecular changes were accompanied by alterations in the local metabolic environment. Pons tissue lactate levels showed a significant main effect of genotype (Figure 2E; two‐way ANOVA, *F*[1,17] = 9.432, *p* = 0.007, *ηp* ^2^ = 0.357), with higher levels in female APP/PS1 mice compared to female WT controls (*p * = 0.028). Expression of the neuronal lactate transporter monocarboxylate transporter 2 (*MCT2*) also showed a significant sex × genotype interaction (Figure 2F; *F*[1,20] = 11.82, *p* = 0.003, *ηp* ^2^ = 0.371), with female APP/PS1 mice exhibiting higher *MCT2* levels than both male APP/PS1 (*p* = 0.002) and female WT (*p *= 0.033).

To assess potential downstream energetic consequences,^37^ we examined oxidative phosphorylation (OXPHOS) components. *Ndufs8* (Complex I) expression showed significant main effects of sex and genotype, as well as a sex × genotype interaction (Figure 2G; all *p* < 0.05). *Atp5a1* (Complex V) exhibited a similar pattern (Figure 2H; all *p* < 0.05). Other OXPHOS genes (*Sdhb*, *Uqcrc1*, and *Cox5b*) did not show significant main or interaction effects (Figure S11 in supporting information).

Taken together, these findings indicate that female APP/PS1 mice exhibit coordinated increases in lactate, *MCT2*, and select OXPHOS components in the pons. While the cellular sources of lactate in this region cannot be determined from these experiments, these measures reflect sex‐dependent differences in lactate, *MCT2*, and OXPHOS‐associated transcripts in the context of amyloid exposure, without evidence for a uniform change across mitochondrial complexes.

### 3.4 EE reverses pontine astrocytic, inflammatory, and OXPHOS‐associated transcript differences in female APP/PS1 mice

To explore whether the molecular alterations observed in female APP/PS1 mice could be modified by experience‐dependent factors, we implemented an EE paradigm that increases sensory, cognitive, and physical stimulation. EE attenuated the elevations in *GFAP, C3, NF‐κB2, MCT2, Ndufs8*, and *Atp5a1* observed under standard housing, with expression levels no longer differing from WT controls (Figure S12A–C,F–H in supporting information; two‐way ANOVA, *p* > 0.05; Table S3). *BDNF* expression (Figure S12D; two‐way ANOVA, *F*[1,24] = 15.17, *p* = 0.001, *ηp* ^2^ = 0.387), however, retained the same sex‐dependent pattern seen in standard housing, indicating that EE did not modify this measure. *IL‐1β* showed no significant genotype‐ or sex‐dependent differences at baseline (Figure S8G), and EE likewise did not alter its expression (Figure S12E).

Together, these results indicate that EE broadly normalizes several astrocyte‐associated, inflammatory, and mitochondrial gene expression changes in the pons of female APP/PS1 mice. Overall, the data show that EE can mitigate multiple pontine molecular alterations associated with early APP/PS1 pathology in a sex‐inclusive manner, without implying a specific cellular mechanism.

### 3.5 LC‐selective GFAP upregulation without evidence of reactive gliosis in female APP/PS1 mice

**FIGURE 3:**
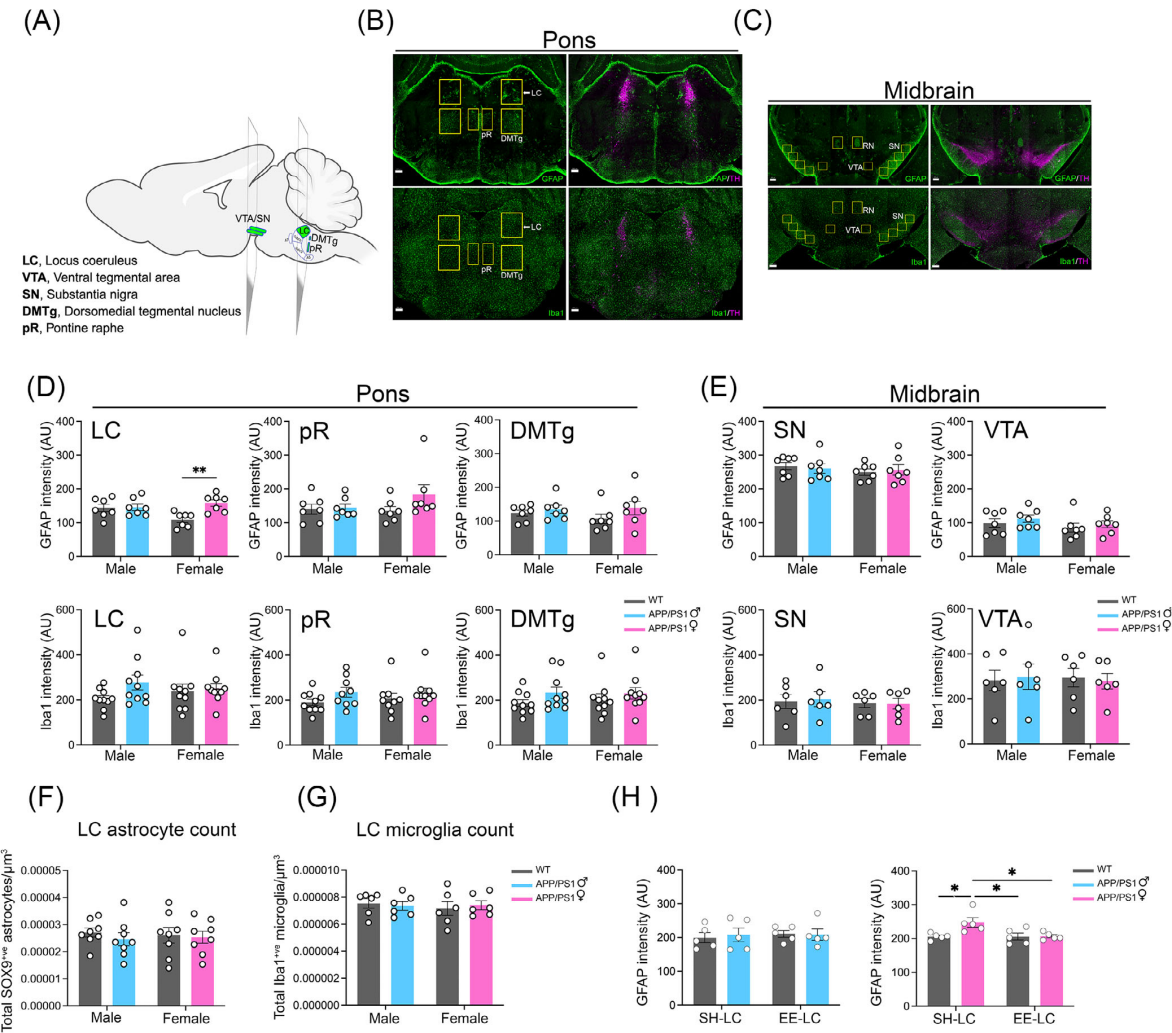
LC‐specific GFAP^+ve^ astrocyte activation in 2‐ to 3‐month‐old female APP/PS1 mice. Immunohistochemical analysis of GFAP^+ve^ astrocytes and Iba1^+ve^ microglia in the pons and midbrain of male and female WT and APP/PS1 mice. A, Schematic showing the anatomical locations of neuromodulatory nuclei—LC, pR, DMTg, SN, and VTA—in the mouse brain. Transparent planes indicate approximate coronal slice levels. Schematic created using Biorender. B, C, Representative confocal images of GFAP^+ve^ astrocytes (green, upper panel) and Iba1^+ve^ microglia (green, lower panel), counterstained with TH (magenta). Yellow boxes mark regions of interest (ROIs) including the LC (marked with white arrow), pR, and DMTg in the pons, and the SN, VTA, and RN in the midbrain. Scale bar = 200 µm. D, LC‐specific pontine GFAP upregulation. A selective increase in GFAP^+ve^ expression was detected in the LC of female APP/PS1 mice compared to female WT (*n* = 7 per group; two‐way ANOVA with Tukey post hoc test; data shown as mean ± SEM; *p < 0.05, **p < 0.01). No significant changes were observed in Iba1^+ve^ microglial expression (*n* = 10 per group in pons; *n* = 6 per group in midbrain; two‐way ANOVA with Tukey post hoc test; data shown as mean ± SEM). No alterations in GFAP^+ve^ astrocyte or Iba1^+ve^ microglial expression were detected in the pR, DMTg, SN, or VTA. F, Estimation of total SOX9^+ve^ astrocytes (*n* = 8 per group; two‐way ANOVA with Tukey post hoc test; data shown as mean ± SEM) and (G) Iba1^+ve^ microglia in the LC (*n* = 6; two‐way ANOVA with Tukey post hoc test; data shown as mean ± SEM). No significant differences in overall glial counts were observed. H, EE normalizes LC‐specific GFAP upregulation in female APP/PS1 mice. Selective increase in GFAP^+ve^ astrocytes detected in the LC of female APP/PS1 mice is normalized to SH baseline upon EE (*n* = 5 per group; two‐way ANOVA with Tukey post hoc test; data shown as mean ± SEM; *p < 0.05). ANOVA, analysis of variance; DMTg, dorsomedial tegmental nucleus; EE, environmental enrichment; GFAP, glial fibrillary acidic protein; Iba1, ionized calcium‐binding adaptor molecule 1; LC, locus coeruleus; pR, pontine raphe; SEM, standard error of mean; SH, standard housing; SN, substantia nigra; SOX9, SRY‐box transcription factor 9; TH, tyrosine hydroxylase; VTA, ventral tegmental area; WT, wild type.

We next assessed region‐specific GFAP expression in the midbrain and pontine neuromodulatory nuclei of 2‐ to 3‐month‐old female APP/PS1 mice (Figure 3A−C). Among the regions examined, the LC showed the most pronounced change, with a significant main effect of genotype (Figure 3D; two‐way ANOVA, *F*[1,24] = 7.344, *p* = 0.012, *ηp* ^2^ = 0.234). A Tukey post hoc test indicated higher GFAP intensity in female APP/PS1 mice compared to female WT controls (*p* = 0.007). Iba1 levels were unchanged, indicating that the LC showed selective GFAP elevation without parallel microglial changes.

In contrast, GFAP and Iba1 intensities in the pontine raphe nucleus (pR), dorsomedial tegmental nucleus (DMTg), substantia nigra (SN), and ventral tegmental area (VTA) did not differ across groups (Figure 3D,E; two‐way ANOVA, *p* > 0.05; Table S3). The red nucleus (RN), included as a non‐neuromodulatory midbrain control, likewise showed no genotype‐ or sex‐related differences (Figure S13 in supporting information; two‐way ANOVA, *p* > 0.05; Table S3).

To evaluate whether the LC GFAP increase reflected changes in astrocyte cell number, we quantified total astrocytes (Sox9^+ve^) and additionally quantified microglia (Iba1^+ve^) cell number. Neither population differed between groups (Figure 3F, G; two‐way ANOVA, *p* > 0.05; Table S3). Sholl analysis of LC GFAP^+ve^ astrocytes revealed no alterations in branching or overall morphology (Figure S14 in supporting information; Mann–Whitney *U* test, *p* > 0.05). These data indicate that elevated GFAP in the LC occurs without detectable changes in astrocyte cell number or structure.

Given the functional relevance of the LC, which provides widespread noradrenergic input to forebrain regions involved in arousal, attention, and memory, this region‐specific GFAP increase may represent an early molecular alteration preceding broader gliosis. EE reduced LC GFAP intensity in female APP/PS1, supported by a significant housing × genotype interaction (Figure 3H; two‐way ANOVA, *F*[1,16] = 4.966, *p* = 0.041, *ηp* ^2^ = 0.237). Tukey post hoc confirmed a decrease in GFAP levels in female APP/PS1 mice housed in EE compared to standard conditions (*p* = 0.029), indicating that this early GFAP elevation is sensitive to environmental modulation.

### 3.6 Sex‐divergent regulation of LC NE‐related markers in 2‐ to 3‐month‐old APP/PS1 mice

**FIGURE 4:**
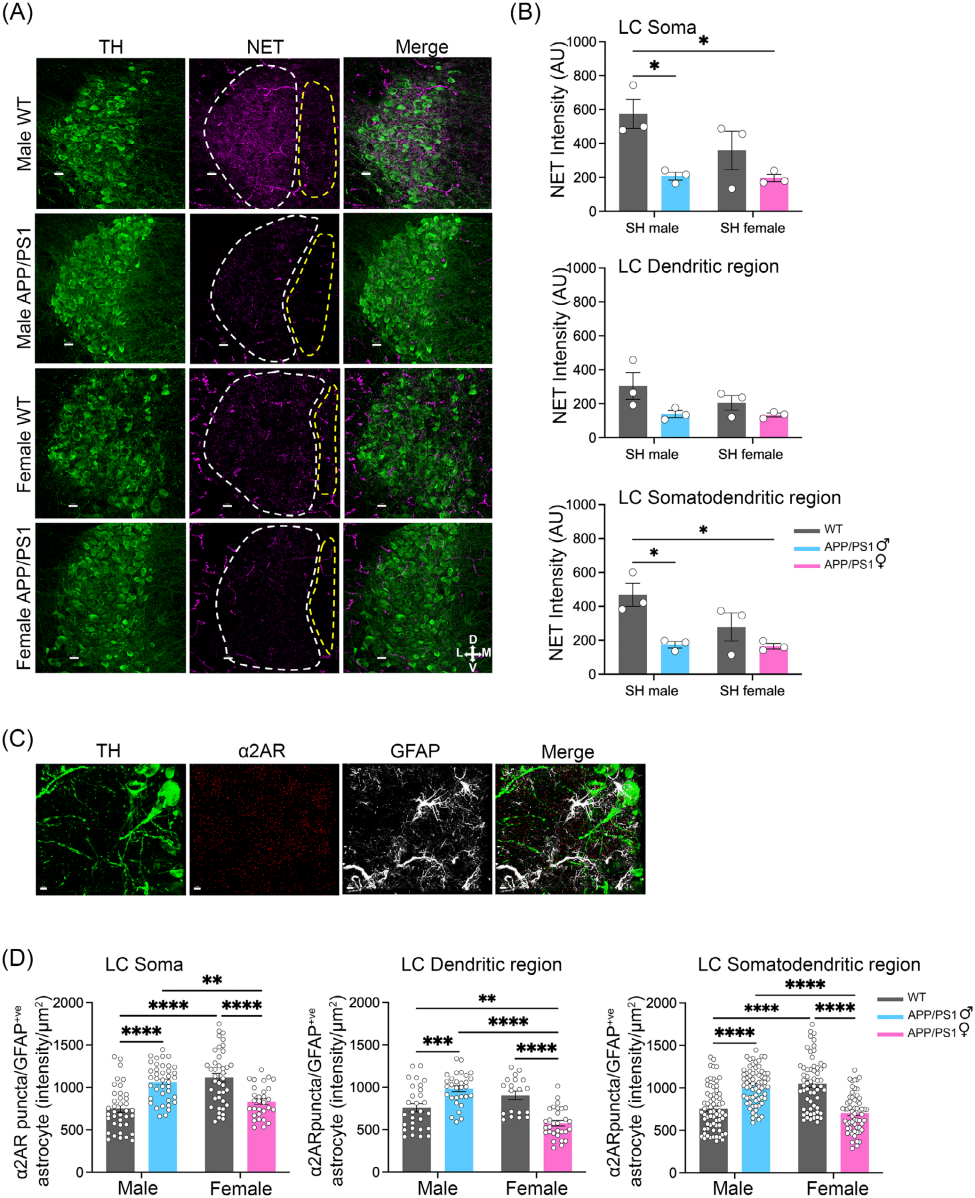
Divergent sex‐specific NE regulation in the LC of 2‐ to 3‐month‐old APP/PS1 mice. A, Representative confocal images of LC soma and dendrites stained for TH (green) and NET (magenta). Scale bar = 20 µm. The dashed white line marks the LC somatic region, and the dashed yellow line marks the medial periLC dendritic region. The anatomical orientation of the LC is shown in the final image of the last row. B, Quantification of NET immunofluorescence in LC soma, dendritic, and somatodendritic regions. Male APP/PS1 mice exhibited a significant reduction in NET levels within the LC somatodendritic region, whereas female APP/PS1 mice showed only a non‐significant decrease compared to their respective WT controls (*n* = 3 per group; two‐way ANOVA with Tukey post hoc test; data presented as mean ± SEM; *p < 0.05). C, Representative confocal images of LC soma and dendrites stained for TH (green), α2AR (red), and GFAP^+ve^ astrocytes (white). Scale bar = 5 µm. D, Quantification of per‐astrocyte α2AR density in the LC soma (astrocytes analyzed: male WT 38, male APP/PS1 39, female WT 40, female APP/PS1 30), LC dendritic region (male WT 29, male APP/PS1 30, female WT 19, female APP/PS1 30), and the combined somatodendritic region (male WT 67, male APP/PS1 69, female WT 59, female APP/PS1 60; *n* = 3–4 mice per group). Male and female APP/PS1 mice exhibited opposite adaptations: astrocytic α2AR was significantly increased in males and significantly decreased in females compared to their respective WT controls. Two‐way ANOVA with Tukey post hoc test; data shown as mean ± SEM; **p < 0.01, ***p < 0.001, ****p < 0.0001. α2AR, alpha 2A adrenergic receptor; ANOVA, analysis of variance; GFAP, glial fibrillary acidic protein; LC, locus coeruleus; NET, norepinephrine transporter; SEM, standard error of mean; TH, tyrosine hydroxylase; WT, wild type.

We next examined LC markers related to NE handling to determine whether sex‐dependent differences were present at this early stage. In the LC somatodendritic region, NET immunoreactivity showed a significant main effect of genotype (Figure 4A,B; two‐way ANOVA, *F*[1,8] = 13.91, *p* = 0.006, *ηp* ^2^ = 0.635). A Tukey post hoc test indicated reduced NET levels in male APP/PS1 mice compared to male WT (*p* = 0.022), whereas differences between female APP/PS1 and female WT mice were not significant (*p* = 0.499).

TH immunointensity exhibited a significant main effect of sex (Figure S15A, B in supporting information; two‐way ANOVA, *F*[1,24] = 6.027, *p* = 0.022, *ηp* ^2^ = 0.201), with female APP/PS1 mice showing higher LC TH intensity than male APP/PS1 mice (*p* = 0.021). LC soma volume did not differ across groups (Figure S15A, C; *p *> 0.05). Under EE, both NET and TH levels no longer differed from WT values (Figure S15D–F; two‐way ANOVA, *p* > 0.05; Table S3).

Total α2AR protein levels in the LC did not differ by sex or genotype (Figure S16A, B in supporting information; two‐way ANOVA, *p* > 0.05). However, when α2AR was quantified specifically in GFAP^+ve^ astrocytes, significant sex × genotype interaction effects emerged in LC soma (Figure 4D; *F*[1,143] = 49.02, *p* < 0.0001, *ηp* ^2^ = 0.255), dendritic regions (*F*[1,104] = 43.80, *p* < 0.0001, *ηp* ^2^ = 0.296), and somatodendritic regions (*F*[1,251] = 96.21, *p* < 0.0001, *ηp* ^2^ = 0.277). Tukey post hoc tests showed increased astrocytic α2AR signal in male APP/PS1 mice compared to male WT (*p *< 0.0001) and reduced astrocytic α2AR signal in female APP/PS1 mice compared to female WT (*p* < 0.0001), reflecting opposite directionality across sexes.

EE did not change total α2AR levels (Figure S16C; two‐way ANOVA, *p* > 0.05). Astrocyte‐resolved α2AR measures under EE no longer showed sex‐ or genotype‐dependent differences (Figure S16D; two‐way ANOVA, *p* > 0.05). In dendritic LC regions, however, female APP/PS1 mice exhibited higher astrocytic α2AR after EE compared to standard housing (*p* = 0.031, Tukey post hoc), whereas no significant EE‐related changes were detected in soma or somatodendritic regions (Table S3).

## 4 DISCUSSION

This study delineates an early, LC‐centered astrocyte state in the brainstem of 2‐ to 3‐month‐old APP/PS1 mice, with effects most prominent in females. Multiple lines of evidence indicate that this state is distinct from classical reactive astrogliosis or microglial activation, and instead represents a primed astrocytic profile aligned with sex‐divergent noradrenergic patterns. By resolving sex differences within this framework, our findings indicate that astrocytic priming in female APP/PS1 mice occurs alongside sex‐divergent noradrenergic pathway signatures during early amyloid exposure, a pattern that may have relevance for later LC vulnerability. Because these experiments were performed in 2‐ to 3‐month‐old APP/PS1 mice, a model that recapitulates early amyloid stress but lacks tau pathology or neurodegeneration, our interpretations remain within an amyloid‐centric context. These findings should be viewed as hypothesized contributors to early vulnerability in this amyloid‐driven model, rather than established mechanisms in human preclinical AD.

### 4.1 Astrocytic activation without classical gliosis

The pons of female APP/PS1 mice showed increased *GFAP, C3*, and *NF‐κB2*, without induction of broader pan‐reactive markers (*vimentin, Serpina3n*, and *S100a10*) and without changes in microglial indices. Morphological analyses further indicated that astrocytes did not undergo hypertrophy, and total astrocyte (Sox9^+ve^) and microglial (Iba1^+ve^) counts were unchanged. GFAP upregulation was anatomically restricted to the LC and did not extend to neighboring monoaminergic nuclei, indicating a region‐selective astrocyte shift rather than global brainstem gliosis.

Female APP/PS1 mice showed higher levels of brainstem Aβ42 oligomers alongside a ^1^H‐MRS profile indicating alterations in metabolites enriched in astrocyte‐related metabolic pathways (Gln, mI, GSH, and Taurine), while neuronal integrity markers (NAA+NAAG, GPC+PCh) were unchanged. The pons also showed increased lactate and elevated *MCT2* expression, indicating shifts in astrocyte–neuron metabolic coupling rather than overt dysfunction. These findings are most consistent with an early, LC‐restricted astrocytic primed state.

### 4.2 NF‐κB2/complement axis and astrocytic priming

The molecular profile in female APP/PS1 mice is consistent with NF‐κB complement–associated signaling within pontine astrocytes. Soluble Aβ oligomers can trigger β1‐integrin/NOX2‐dependent reactive oxygen species generation and subsequent NF‐κB activation in astrocytes,^13^, ^38^, ^39^ consistent with elevated *GFAP, C3*, and *NF‐κB2* observed here. The rise in *NF‐κB2* suggests partial engagement of the non‐canonical pathway described in astrocytic inflammatory responses.^40^, ^41^ Supporting this interpretation, MRS‐detected increases in GSH, together with higher lactate levels, increased *MCT2* expression, and elevations in selected OXPHOS‐associated transcripts (*Ndufs8, Atp5a1*), reflect coordinated differences in antioxidant‐related metabolites and lactate–OXPHOS–linked gene expression rather than uniform changes across mitochondrial complexes.^42^

Although C3 is a hallmark of A1‐type astrocytes,^43^, ^44^ the accompanying increase in GFAP occurs without additional A1 markers, astrocyte hypertrophy, or microglial activation, indicating an early, primed, non‐reactive state rather than a mature neurotoxic phenotype. This intermediate profile may help maintain local stability under low‐level oligomeric stress while potentially lowering the threshold for later activation.

### 4.3 Astrocyte‐first, microglia‐sparing sequence

Our results support an astrocyte‐first response to soluble Aβ oligomers. In AppNL‐G‐F mice astrocytosis emerges before microgliosis and becomes prominent only after plaque accrual,^45^ and oligomeric Aβ can directly activate astrocytes even in neuron‐free systems.^46^ Single‐cell atlases identify astrocytic programs that precede or dominate microglial signatures during early Aβ exposure.^47^, ^48^ Human cerebrospinal fluid and brain tissue likewise exhibit early complement activation,^49^, ^50^ and ^1^H‐MRS markers such as elevated mI, with accompanying Gln and GSH shifts, map to astrocyte remodeling in prodromal cohorts.^51^ Consistent with this framework, the ACSA‐1–enriched astrocyte fractions exhibited higher *C3* levels, indicating that the enrichment protocol captures *C3*‐expressing cells; however, because the samples were pooled, the magnitude of astrocytic contribution cannot be determined. Collectively, these data point toward oligomer‐associated, astrocyte‐linked molecular changes.

### 4.4 LC‐related molecular differences and sex‐biased patterns

The LC is among the earliest sites of tau pathology in AD,^5^, ^6^ but APP/PS1 mice lack tau aggregates, and show intact LC neurons at 3 months; the changes observed here are therefore amyloid driven. The selective GFAP elevation in female LC, alongside preserved neuronal integrity, suggests an early astrocyte state. Human imaging and behavioral studies similarly link preserved LC‐linked biochemical measures and short‐term cognitive performance in early amyloid contexts.^25^, ^52^, ^53^ This pattern may reflect an early, sex‐linked astrocytic state with potential relevance to LC vulnerability.^54^, ^55^

### 4.5 LC‐first vulnerability and potential points of modulation

Recent transcriptomic comparisons between the vulnerable LC and the relatively resilient SN provide broader context. LC neurons exhibit higher low‐density lipoprotein receptor (LDLR)/transmembrane protein 97 (TMEM97) and lower myosin regulatory light chain interacting protein/increased degradation of LDL receptor protein, suggesting differences in lipid–apolipoprotein E (apoE)‐related pathways that may influence Aβ oligomer handling.^56^ SN enrichment for antioxidant/metallothionein pathways may buffer stress more effectively. *Post mortem* and mouse studies confirm Aβ oligomer accumulation in LC neurons, impairing GABA/glycine receptor function and increasing excitability.^57^ Within this framework, the LC‐adjacent astrocytic profile identified here—GFAP elevation without classical reactivity—may represent an early molecular state with potential relevance to local vulnerability, although its mechanistic significance remains hypothetical. Literature has proposed that modulating apoE–LDLR/TMEM97 pathways or supporting antioxidant capacity could represent points of intervention, but how these relate to the current findings requires further study.

### 4.6 Early LC GFAP changes relative to plasma markers

Plasma GFAP is a sensitive biomarker of AD but typically increases only when astrocytes become reactive and cytoskeletal integrity is perturbed.^10^, ^58^ In the present study, we observed a modest, pre‐reactive increase in LC GFAP at 2 to 3 months, a stage that precedes overt gliosis in this model. Although direct correspondence to human biomarker trajectories cannot be assumed, these findings raise the hypothesis that LC‐localized astrocytic changes may occur before detectable plasma GFAP alterations. Establishing whether such early LC signals genuinely precede or predict peripheral biomarker changes will require longitudinal and cross‐species studies, but the current data suggest a potential cellular event earlier in the astrocytic response sequence.

### 4.7 EE and restoration of NE tone

EE is known to enhance central noradrenergic signaling and mitigate amyloid‐associated glial activation in several AD models, including effects mediated through β‐adrenergic pathways.^59^, ^60^ In our study, EE similarly reduced LC‐centered astrocyte priming, normalizing *GFAP, C3, NF‐κB2*, and MCT2−OXPHOS‐associated transcripts in female APP/PS1 mice. These changes are consistent with a shift toward normalization of NE‐related protein signatures, and decreased inflammatory load within the LC microenvironment. Notably, EE did not alter *BDNF* levels, suggesting that its effects at this early stage act primarily through modulation of astrocyte–NE coupling rather than neurotrophic pathways.

### 4.8 Sex‐divergent LC noradrenergic tuning

Our findings identify an early, sex‐divergent shift in LC noradrenergic regulation within this amyloid‐driven model. In males, reduced NET together with elevated astrocytic α2AR resembles configurations previously associated with prolonged extracellular NE and stronger Gi/o‐mediated astrocytic inhibition—conditions thought to stabilize local excitability and moderate inflammatory signaling.^61^, ^62^ In females, increased TH together with reduced astrocytic α2AR forms a contrasting receptor profile, consistent with reports of weaker NE‐dependent regulatory feedback in comparable contexts.^63^, ^64^ Such α2AR reduction may render female astrocytes less responsive to NE, potentially diminishing inhibitory control over local inflammatory signaling—a hypothesis aligned with the stronger astrocytic priming observed in females. Under early Aβ exposure—shown to heighten LC excitability in prior work^57^, ^65^—these sex‐divergent receptor patterns may help explain differential LC susceptibility.

EE restored NET and α2AR expression overall, indicating that these early noradrenergic–astrocytic interactions remain plastic. In female APP/PS1 mice, EE increased astrocytic α2AR above baseline, suggesting strengthened NE–astrocyte inhibitory coupling rather than simple rescue. These effects parallel prior work showing that increasing NE tone can dampen glial activation in amyloid models.^66^, ^67^ Human studies likewise report early LC alterations—elevated LC contrast, NE dysregulation, and molecular stress signatures—before structural degeneration.^19^, ^25^, ^68^, ^69^ The sex‐biased astrocytic and noradrenergic patterns identified here align with these early‐stage observations while remaining constrained by the amyloid‐only nature of the APP/PS1 model.

### 4.9 NF‐κB–BACE1 axis and amyloidogenic drive

The NF‐κB–BACE1 axis has been described in other models as linking inflammatory signaling to amyloidogenic processes.^70^, ^71^ Increased *BACE1* expression in female APP/PS1 mice is consistent with these reports, but the mechanistic relevance of this axis in the present study remains hypothetical.

### 4.10 Translational relevance and sex‐specific biomarkers

Human data increasingly show sex‐dependent patterns in astrocyte‐linked biomarkers, with GFAP, chitinase‐3‐like protein 1, and sTREM2 displaying stronger associations with amyloid/tau and atrophy in unimpaired adults at risk for AD.^72^ Plasma GFAP trajectories also differ by sex, with women showing higher levels and stronger links to neurodegeneration,^73^, ^74^ and imaging studies suggesting that GFAP may index microstructural vulnerability differently across sexes.^75^ Although we did not measure biomarkers directly, the modest, non‐reactive GFAP increase in female LC astrocytes raises the possibility that early sex‐biased astrocytic states may underlie later divergence in biomarker trajectories. This remains speculative but underscores the importance of sex‐aware interpretation.

### 4.11 Limitations

APP/PS1 mice at 2 to 3 months lack tau pathology and neurodegeneration, reflecting an amyloid‐driven state that differs from astrocytic profiles in aged or tau‐bearing brains. As a familial, amyloid‐only model, it also fails to capture the heterogeneity of sporadic AD—including interactions among amyloid, tau, aging, and systemic influences. Methodological constraints—including MRS voxels spanning multiple nuclei, immunohistochemical rather than functional assessment of NET and α2AR, and the multifactorial nature of EE—limit mechanistic inference. Finally, the cross‐sectional design precludes resolving temporal ordering or causal relationships.

## CONFLICT OF INTEREST STATEMENT

The authors declare no conflicts of interest. Author disclosures are available in the supporting information.

## CONSENT STATEMENT

No human subjects were used for the present study. Therefore, consent was not necessary.

## Supporting information



Supporting Information

Supporting Information

Supporting Information

Supporting Information

Supporting Information

Supporting Information

Supporting Information

Supporting Information

Supporting Information

Supporting Information

Supporting Information

Supporting Information

Supporting Information

Supporting Information

Supporting Information

Supporting Information

Supporting Information

Supporting Information

Supporting Information

Supporting Information
