# Supplementary material for: Female‐biased astrocytic priming shapes early locus coeruleus vulnerability in an Aβ oligomer milieu
Source: Alzheimers Dement. 2026 Feb 6;22(2):e71168. doi: 10.1002/alz.71168 (PMC12877963; doi:10.1002/alz.71168)
Supplement: Supplementary file 1 — Supporting Information [file ALZ-22-e71168-s004.docx]

| **Target** | **Forward** | **Reverse** |
| --- | --- | --- |
| *Gfap* | 5’-AGAACAACCTGGCTGCGTAT-3’ | 5’-CTTGGCCACATCCATCTCCA-3’ |
| *C3* | 5’-AAGCATCAACACACCCAACA-3’ | 5’-CTTGAGCTCCATTCGTGACA-3’ |
| *Tgf-β* | 5’-CCGCAACAACGCCATCTATG-3’ | 5’-CCCGAATGTCTGACGTATTGAAG-3’ |
| *S100a10* | 5’-CCTCTGGCTGTGGACAAAAT-3’ | 5’-CTGCTCACAAGAAGCAGTGG-3’ |
| *Il-1β* | 5’-GCAACTGTTCCTGAACTCAACT-3’ | 5’-ATCTTTTGGGGTCCGTCAACT-3’ |
| *Tnf-α* | 5’-GGCAGGTTCTGTCCCTTTCAC-3’ | 5’-TTCTGTGCTCATGGTGTCTTTTCT-3’ |
| *Nos2* | 5’-GTTCTCAGCCCAACAATACAAGA-3’ | 5’-GTGGACGGGTCGATGTCAC-3’ |
| *Cd68* | 5’-TGTCTGATCTTGCTAGGACCG-3’ | 5’-GAGAGTAACGGCCTTTTTGTGA-3’ |
| *C1q* | 5’-AAAGGCAATCCAGGCAATATCA-3’ | 5’-TGGTTCTGGTATGGACTCTCC-3’ |
| *Cx3Cr1* | 5’-GAGTATGACGATTCTGCTGAGG-3’ | 5’-CAGACCGAACGTGAAGACGAG-3’ |
| *Il-6* | 5’-GTTGCCTTCTTGGGACTGATG-3’ | 5’-TGGGAGTGGTATCCTCTGTGAA-3’ |
| *Cox2* | 5’-CATGGACTCACTCAGTTTGTT-3’ | 5’-GAAGCGTTTGCGGTACTCATT-3’ |
| *Nfκb1* | 5’-GAGTACGACAACATCTCCTTG-3’ | 5’-CAGAGGTGTAGTCCCATCATA-3’ |
| *Nfκb2* | 5’-CTGGTGGACACATACAGGAAGAC-3’ | 5’-ATAGGCACTGTCTTCTTTCACCTC-3’ |
| *Bdnf* | 5’-GCCTTTGGAGCCTCCTCTAC-3’ | 5’-GCGGCATCCAGGTAATTTT-3’ |
| *Vimentin* | 5’-ATGCTTCTCTGGCACGTCTT-3’ | 5’-AGCCACGCTTTCATACTGCT-3’ |
| *Serpina3n* | 5’-ATTTGTCCCAATGTCTGCGAA-3’ | 5’-TGGCTATCTTGGCTATAAAGGGG-3’ |
| *Ndufs8* | 5’-TGGCGGCAACGTACAAGTAT-3’ | 5’-GTAGTTGATGGTGGCAGGCT-3’ |
| *Sdhb* | 5’-TCGAATGCAGACGTACGAGG-3’ | 5’-AAGAGCCACAGATGCCTTCTC-3’ |
| *Uqcrc1* | 5’-ACTCGGGGCAAAAACATCCT-3’ | 5’-GCAAATGTCACGCAGCATCT-3’ |
| *Cox5b* | 5’-GTTAGACTCCCACCAACGGA-3’ | 5’-ACTCCGCGAAGTAACCTTGA-3’ |
| *Atp5a1* | 5’-CGGCCATTTTGTGCCAGTC-3’ | 5’-CCAAAGCATTTTTGGAGACCAGT-3’ |
| *Mct2* | 5’-TGTATGCGGGAGGTCCCAT-3’ | 5’-AGGCTGGTTGCAGGTTGAAT-3’ |
| *Bace1* | 5’-GGAACCCATCTCGGCATCC-3’ | 5’-TCCGATTCCTCGTCGGTCTC-3’ |
| *18S rRNA* | 5’-TTTCGAGGCCCTGTAATTGG-3’ | 5’-CCCAAGATCCAACTACGAGC-3’ |

# SUPPLEMENTARY TABLE S1: The list of primer sequences used for qPCR
