# Supplementary material for: Female‐biased astrocytic priming shapes early locus coeruleus vulnerability in an Aβ oligomer milieu
Source: Alzheimers Dement. 2026 Feb 6;22(2):e71168. doi: 10.1002/alz.71168 (PMC12877963; doi:10.1002/alz.71168)
Supplement: Supplementary file 2 — Supporting Information [file ALZ-22-e71168-s005.docx]

SUPPLEMENTARY TABLE S2: Relative concentration ratios of brainstem metabolites per total creatine (Cr+PCr) in 2-3-month-old male and female WT and APP/PS1 mice

| Genotype | Male WT | Male APP/PS1 | Female WT | Female APP/PS1 |
| --- | --- | --- | --- | --- |
| Gln | 2.47±0.28 | 2.54±0.24 | 2.29±0.16 | 3.31±0.2 |
| Glu | 5.31±0.55 | 5.14±0.72 | 4.64±0.66 | 6.9±0.41 |
| Glx | 7.77±0.81 | 7.69±0.91 | 6.93±0.75 | 10.2±0.38 |
| GSH | 1.81±0.18 | 1.42±0.15 | 1.41±0.1 | 2.03±0.18 |
| mI | 6.73±0.6 | 5.95±0.47 | 6.38±0.46 | 8.55±0.56 |
| Taurine | 3.96±0.64 | 3.51±0.34 | 3.03±0.42 | 5.14±0.3 |
| NAA | 4.7±0.35 | 3.81±0.49 | 4.06±0.68 | 5.46±0.39 |
| NAA+NAAG | 5.06±0.29 | 4.96±0.7 | 5.01±0.67 | 6.18±0.35 |
| GPC+PCh | 1.18±0.1 | 1.25±0.19 | 1.32±0.15 | 1.45±0.12 |
| Cr+PCr | 6.19±0.77 | 5.71±0.48 | 5.79±0.59 | 7.69±0.23 |

Note: All data are represented as Mean ± SEM.

**Abbreviations:** Cr+PCr; creatine+phosphocreatine Gln, glutamine; Glu, glutamate; Glx, glutamine/glutamate; GPC+PCh, glycerophosphocholine+phosphocholine; GSH, glutathione; mI, myo-inositol; NAA, N-acetyl aspartate; NAA+NAAG, N-Acetylaspartylglutamate; SEM, standard error of mean; WT, wild-type.
