# Supplementary material for: Female‐biased astrocytic priming shapes early locus coeruleus vulnerability in an Aβ oligomer milieu
Source: Alzheimers Dement. 2026 Feb 6;22(2):e71168. doi: 10.1002/alz.71168 (PMC12877963; doi:10.1002/alz.71168)
Supplement: Supplementary file 3 — Supporting Information [file ALZ-22-e71168-s014.docx]

# SUPPLEMENTARY TABLE S3: Details of statistical analysis

| **Figure Number** | **Test Performed** | **Number of animals (Male / Female) per genotype or group sizes** | **Effect** | **Test Statistic (degrees of freedom)** | ***p* value** |
| --- | --- | --- | --- | --- | --- |
| **Figure 1A** |  |  |  |  |  |
| Gln | Two-way ANOVA (sex x genotype) | WT: 6/7  APP/PS1:8/5 | Sex | F (1, 22) = 1.651 | 0.2121 |
|  |  |  | Genotype | F (1, 22) = 5.534 | 0.0280 |
|  |  |  | Interaction | F (1, 22) = 4.097 | 0.0553 |
| **Figure 1B** |  |  |  |  |  |
| GSH | Two-way ANOVA (sex x genotype) | WT: 5/7  APP/PS1:8/5 | Sex | F (1, 21) = 0.4204 | 0.5238 |
|  |  |  | Genotype | F (1, 21) = 0.5684 | 0.4593 |
|  |  |  | Interaction | F (1, 21) = 10.85 | 0.0035 |
| **Figure 1C** |  |  |  |  |  |
| mI | Two-way ANOVA (sex x genotype) | WT: 6/7  APP/PS1:8/5 | Sex | F (1, 22) = 4.594 | 0.0434 |
|  |  |  | Genotype | F (1, 22) = 1.757 | 0.1987 |
|  |  |  | Interaction | F (1, 22) = 7.853 | 0.0104 |
| **Figure 1D** |  |  |  |  |  |
| Taurine | Two-way ANOVA (sex x genotype) | WT: 6/6  APP/PS1:8/5 | Sex | F (1, 21) = 0.6069 | 0.4446 |
|  |  |  | Genotype | F (1, 21) = 3.404 | 0.0792 |
|  |  |  | Interaction | F (1, 21) = 8.079 | 0.0098 |
| **Figure 1F** |  |  |  |  |  |
| GFAP | Two-way ANOVA (sex x genotype) | WT: 4/5  APP/PS1:5/5 | Sex | F (1, 15) = 7.670 | 0.0143 |
|  |  |  | Genotype | F (1, 15) = 4.121 | 0.0605 |
|  |  |  | Interaction | F (1, 15) = 8.460 | 0.0108 |
| **Figure 1G** |  |  |  |  |  |
| C3 | Two-way ANOVA (sex x genotype) | WT: 6/5  APP/PS1:6/6 | Sex | F (1, 19) = 2.832 | 0.1088 |
|  |  |  | Genotype | F (1, 19) = 3.934 | 0.0620 |
|  |  |  | Interaction | F (1, 19) = 4.711 | 0.0428 |
| **Figure 1H** |  |  |  |  |  |
| NFκB2 | Two-way ANOVA (sex x genotype) | WT: 6/6  APP/PS1:6/6 | Sex | F (1, 20) = 9.340 | 0.0062 |
|  |  |  | Genotype | F (1, 20) = 28.58 | <0.0001 |
|  |  |  | Interaction | F (1, 20) = 3.020 | 0.0976 |
| **Figure 1I** |  |  |  |  |  |
| BDNF | Two-way ANOVA (sex x genotype) | WT: 6/6  APP/PS1:6/6 | Sex | F (1, 20) = 11.63 | 0.0028 |
|  |  |  | Genotype | F (1, 20) = 2.284 | 0.1464 |
|  |  |  | Interaction | F (1, 20) = 0.9539 | 0.3404 |
| **Figure 2E** |  |  |  |  |  |
| Lactate | Two-way ANOVA (sex x genotype) | WT: 5/6  APP/PS1:5/5 | Sex | F (1, 17) = 2.218 | 0.1547 |
|  |  |  | Genotype | F (1, 17) = 9.432 | 0.0069 |
|  |  |  | Interaction | F (1, 17) = 1.586 | 0.2250 |
| **Figure 2F** |  |  |  |  |  |
| MCT2 | Two-way ANOVA (sex x genotype) | WT: 6/6  APP/PS1:6/6 | Sex | F (1, 20) = 6.068 | 0.0230 |
|  |  |  | Genotype | F (1, 20) = 0.6545 | 0.4280 |
|  |  |  | Interaction | F (1, 20) = 11.82 | 0.0026 |
| **Figure 2G** |  |  |  |  |  |
| Ndufs8 | Two-way ANOVA (sex x genotype) | WT: 6/6  APP/PS1:5/6 | Sex | F (1, 19) = 6.423 | 0.0202 |
|  |  |  | Genotype | F (1, 19) = 4.474 | 0.0479 |
|  |  |  | Interaction | F (1, 19) = 8.960 | 0.0075 |
| **Figure 2H** |  |  |  |  |  |
| Atp5a1 | Two-way ANOVA (sex x genotype) | WT: 6/6  APP/PS1:6/5 | Sex | F (1, 19) = 9.071 | 0.0072 |
|  |  |  | Genotype | F (1, 19) = 8.020 | 0.0107 |
|  |  |  | Interaction | F (1, 19) = 4.412 | 0.0493 |
| **Figure 3D** |  |  |  |  |  |
| GFAP-LC | Two-way ANOVA (sex x genotype) | WT: 7/7  APP/PS1:7/7 | Sex | F (1, 24) = 1.314 | 0.2629 |
|  |  |  | Genotype | F (1, 24) = 7.344 | 0.0122 |
|  |  |  | Interaction | F (1, 24) = 5.918 | 0.0228 |
| GFAP-pR | Two-way ANOVA (sex x genotype) | WT: 7/7  APP/PS1:7/7 | Sex | F (1, 24) = 0.9016 | 0.3518 |
|  |  |  | Genotype | F (1, 24) = 2.008 | 0.1693 |
|  |  |  | Interaction | F (1, 24) = 1.418 | 0.2453 |
| GFAP-DMTg | Two-way ANOVA (sex x genotype) | WT: 7/7  APP/PS1:7/7 | Sex | F (1, 24) = 0.2089 | 0.6518 |
|  |  |  | Genotype | F (1, 24) = 2.187 | 0.1522 |
|  |  |  | Interaction | F (1, 24) = 0.4902 | 0.4906 |
| Iba1-LC | Two-way ANOVA (sex x genotype) | WT: 10/10  APP/PS1:10/10 | Sex | F (1, 36) = 0.01714 | 0.8966 |
|  |  |  | Genotype | F (1, 36) = 2.532 | 0.1203 |
|  |  |  | Interaction | F (1, 36) = 1.257 | 0.2696 |
| Iba1-pR | Two-way ANOVA (sex x genotype) | WT: 10/10  APP/PS1:10/10 | Sex | F (1, 36) = 0.06184 | 0.8051 |
|  |  |  | Genotype | F (1, 36) = 2.552 | 0.1194 |
|  |  |  | Interaction | F (1, 36) = 0.3228 | 0.5737 |
| Iba1-DMTg | Two-way ANOVA (sex x genotype) | WT: 10/10  APP/PS1:10/10 | Sex | F (1, 36) = 0.09707 | 0.7572 |
|  |  |  | Genotype | F (1, 36) = 2.517 | 0.1214 |
|  |  |  | Interaction | F (1, 36) = 0.1890 | 0.6663 |
| **Figure 3E** |  |  |  |  |  |
| GFAP-SN | Two-way ANOVA (sex x genotype) | WT: 7/7  APP/PS1:7/7 | Sex | F (1, 24) = 0.7840 | 0.3847 |
|  |  |  | Genotype | F (1, 24) = 0.0006322 | 0.9801 |
|  |  |  | Interaction | F (1, 24) = 0.3043 | 0.5863 |
| GFAP-VTA | Two-way ANOVA (sex x genotype) | WT: 7/7  APP/PS1:7/7 | Sex | F (1, 24) = 1.583 | 0.2204 |
|  |  |  | Genotype | F (1, 24) = 0.9291 | 0.3447 |
|  |  |  | Interaction | F (1, 24) = 0.01661 | 0.8985 |
| Iba1-SN | Two-way ANOVA (sex x genotype) | WT: 6/6  APP/PS1:6/6 | Sex | F (1, 20) = 0.2317 | 0.6355 |
|  |  |  | Genotype | F (1, 20) = 0.01904 | 0.8916 |
|  |  |  | Interaction | F (1, 20) = 0.05552 | 0.8161 |
| Iba1-VTA | Two-way ANOVA (sex x genotype) | WT: 6/6  APP/PS1:6/6 | Sex | F (1, 20) = 0.006843 | 0.9349 |
|  |  |  | Genotype | F (1, 20) = 1.492e-005 | 0.9970 |
|  |  |  | Interaction | F (1, 20) = 0.1265 | 0.7258 |
| **Figure 3F** |  |  |  |  |  |
| LC astrocyte count (Sox9^+ve^) | Two-way ANOVA (sex x genotype) | WT: 8/8  APP/PS1:8/8 | Sex | F (1, 28) = 0.002915 | 0.9573 |
|  |  |  | Genotype | F (1, 28) = 0.4601 | 0.5031 |
|  |  |  | Interaction | F (1, 28) = 0.1556 | 0.6963 |
| **Figure 3G** |  |  |  |  |  |
| LC microglia count  (Iba1^+ve^) | Two-way ANOVA (sex x genotype) | WT: 6/6  APP/PS1:6/6 | Sex | F (1, 20) = 0.1927 | 0.6654 |
|  |  |  | Genotype | F (1, 20) = 0.006059 | 0.9387 |
|  |  |  | Interaction | F (1, 20) = 0.2934 | 0.5940 |
| **Figure 3H** |  |  |  |  |  |
| Male GFAP-LC | Two-way ANOVA (housing x genotype) | WT: 5/5  APP/PS1:5/5 | Housing | F (1, 16) = 0.1422 | 0.7111 |
|  |  |  | Genotype | F (1, 16) = 0.03127 | 0.8619 |
|  |  |  | Interaction | F (1, 16) = 0.1075 | 0.7472 |
| Female GFAP-LC | Two-way ANOVA (housing x genotype) | WT: 5/5  APP/PS1:5/5 | Housing | F (1, 16) = 4.880 | 0.0421 |
|  |  |  | Genotype | F (1, 16) = 5.348 | 0.0344 |
|  |  |  | Interaction | F (1, 16) = 4.966 | 0.0405 |
| **Figure 4B** |  |  |  |  |  |
| LC-NET Soma | Two-way ANOVA (sex x genotype) | WT: 3/3  APP/PS1:3/3 | Sex | F (1, 8) = 2.390 | 0.1607 |
|  |  |  | Genotype | F (1, 8) = 11.77 | 0.0089 |
|  |  |  | Interaction | F (1, 8) = 1.962 | 0.1989 |
| LC-NET Dendritic region | Two-way ANOVA (sex x genotype) | WT: 3/3  APP/PS1:3/3 | Sex | F (1, 8) = 1.262 | 0.2939 |
|  |  |  | Genotype | F (1, 8) = 6.497 | 0.0342 |
|  |  |  | Interaction | F (1, 8) = 1.008 | 0.3448 |
| LC-NET  Somatodendritic region | Two-way ANOVA (sex x genotype) | WT: 3/3  APP/PS1:3/3 | Sex | F (1, 8) = 3.285 | 0.1075 |
|  |  |  | Genotype | F (1, 8) = 13.91 | 0.0058 |
|  |  |  | Interaction | F (1, 8) = 2.757 | 0.1354 |
| **Figure 4D** |  |  |  |  |  |
| LC-Astrocytic α2AR Soma | Two-way ANOVA (sex x genotype) | WT: 3/3  APP/PS1:3/3  (38-40 astrocytes/group) | Sex | F (1, 143) = 2.506 | 0.1156 |
|  |  |  | Genotype | F (1, 143) = 0.1136 | 0.7365 |
|  |  |  | Interaction | F (1, 143) = 49.02 | <0.0001 |
| LC-Astrocytic α2AR Dendritic region | Two-way ANOVA (sex x genotype) | WT: 3/3  APP/PS1:3/3  (19-30 astrocytes/group) | Sex | F (1, 104) = 9.726 | 0.0023 |
|  |  |  | Genotype | F (1, 104) = 1.583 | 0.2111 |
|  |  |  | Interaction | F (1, 104) = 43.80 | <0.0001 |
| LC-Astrocytic α2AR  Somatodendritic region | Two-way ANOVA (sex x genotype) | WT: 3/3  APP/PS1:3/3  (59-69 astrocytes/group) | Sex | F (1, 251) = 0.2230 | 0.6372 |
|  |  |  | Genotype | F (1, 251) = 1.240 | 0.2665 |
|  |  |  | Interaction | F (1, 251) = 96.21 | <0.0001 |
| **Supplementary Figure S5A** |  |  |  |  |  |
| Aβ42 oligomers | Two-way ANOVA (sex x genotype) | WT: 3/3  APP/PS1:5/6 | Sex | F (1, 13) = 5.223 | 0.0397 |
|  |  |  | Genotype | F (1, 13) = 80.01 | <0.0001 |
|  |  |  | Interaction | F (1, 13) = 5.223 | 0.0397 |
| **Supplementary Figure S5B** |  |  |  |  |  |
| BACE1 | Mann-Whitney U test | Male APP/PS1: 6  Female APP/PS1:6 | - | U=5  Median (Male)=1.703  Median (Female)=2.049 | 0.0411 |
| **Supplementary Figure S5C** |  |  |  |  |  |
| Glu | Two-way ANOVA (sex x genotype) | WT: 6/7  APP/PS1:8/5 | Sex | F (1, 22) = 0.6801 | 0.4184 |
|  |  |  | Genotype | F (1, 22) = 2.556 | 0.1241 |
|  |  |  | Interaction | F (1, 22) = 3.402 | 0.0786 |
| **Supplementary Figure S5D** |  |  |  |  |  |
| Glx | Two-way ANOVA (sex x genotype) | WT: 6/7  APP/PS1:8/5 | Sex | F (1, 22) = 1.057 | 0.3150 |
|  |  |  | Genotype | F (1, 22) = 3.815 | 0.0636 |
|  |  |  | Interaction | F (1, 22) = 4.234 | 0.0517 |
| **Supplementary Figure S5E** |  |  |  |  |  |
| NAA | Two-way ANOVA (sex x genotype) | WT: 6/7  APP/PS1:8/5 | Sex | F (1, 22) = 0.8985 | 0.3535 |
|  |  |  | Genotype | F (1, 22) = 0.2306 | 0.6358 |
|  |  |  | Interaction | F (1, 22) = 4.577 | 0.0438 |
| **Supplementary Figure S5F** |  |  |  |  |  |
| NAA+NAAG | Two-way ANOVA (sex x genotype) | WT: 6/7  APP/PS1:8/5 | Sex | F (1, 22) = 0.9159 | 0.3490 |
|  |  |  | Genotype | F (1, 22) = 0.7534 | 0.3948 |
|  |  |  | Interaction | F (1, 22) = 1.087 | 0.3085 |
| **Supplementary Figure S5G** |  |  |  |  |  |
| GPC+PCh | Two-way ANOVA (sex x genotype) | WT: 6/7  APP/PS1:8/5 | Sex | F (1, 22) = 1.065 | 0.3133 |
|  |  |  | Genotype | F (1, 22) = 0.3876 | 0.5400 |
|  |  |  | Interaction | F (1, 22) = 0.03873 | 0.8458 |
| **Supplementary Figure S5H** |  |  |  |  |  |
| Cr+PCr | Two-way ANOVA (sex x genotype) | WT: 6/7  APP/PS1:8/5 | Sex | F (1, 22) = 1.869 | 0.1854 |
|  |  |  | Genotype | F (1, 22) =1.493 | 0.2347 |
|  |  |  | Interaction | F (1, 22) = 4.238 | 0.0516 |
| **Supplementary Figure S6C** |  |  |  |  |  |
| TH | Unpaired t test | Pons:6  Midbrain:6 | - | t (10) = 4.232 | 0.0017 |
| DBH | Unpaired t test | Pons:6  Midbrain:6 | - | t (10) = 2.534 | 0.0296 |
| **Supplementary Figure S7A** |  |  |  |  |  |
| Vimentin | Two-way ANOVA (sex x genotype) | WT: 5/6  APP/PS1:6/6 | Sex | F (1, 19) = 0.6456 | 0.4316 |
|  |  |  | Genotype | F (1, 19) = 0.06958 | 0.7948 |
|  |  |  | Interaction | F (1, 19) = 0.4518 | 0.5096 |
| **Supplementary Figure S7B** |  |  |  |  |  |
| Serpina3n | Two-way ANOVA (sex x genotype) | WT: 6/6  APP/PS1:6/6 | Sex | F (1, 20) = 1.532 | 0.2302 |
|  |  |  | Genotype | F (1, 20) = 0.2496 | 0.6228 |
|  |  |  | Interaction | F (1, 20) = 2.501 | 0.1295 |
| **Supplementary Figure S7C** |  |  |  |  |  |
| S100a10 | Two-way ANOVA (sex x genotype) | WT: 6/6  APP/PS1:6/6 | Sex | F (1, 20) = 2.034 | 0.1692 |
|  |  |  | Genotype | F (1, 20) = 6.276 | 0.0210 |
|  |  |  | Interaction | F (1, 20) = 1.074 | 0.3124 |
| **Supplementary Figure S8A** |  |  |  |  |  |
| TGFβ | Two-way ANOVA (sex x genotype) | WT: 6/6  APP/PS1:6/6 | Sex | F (1, 20) = 2.648 | 0.1193 |
|  |  |  | Genotype | F (1, 20) = 1.902 | 0.1830 |
|  |  |  | Interaction | F (1, 20) = 0.7651 | 0.3921 |
| **Supplementary Figure S8B** |  |  |  |  |  |
| iNOS | Two-way ANOVA (sex x genotype) | WT: 6/6  APP/PS1:6/6 | Sex | F (1, 20) = 3.090 | 0.0941 |
|  |  |  | Genotype | F (1, 20) = 0.6248 | 0.4385 |
|  |  |  | Interaction | F (1, 20) = 0.0001756 | 0.9896 |
| **Supplementary Figure S8C** |  |  |  |  |  |
| CD68 | Two-way ANOVA (sex x genotype) | WT: 6/6  APP/PS1:6/6 | Sex | F (1, 20) = 2.724 | 0.1145 |
|  |  |  | Genotype | F (1, 20) = 4.588 | 0.0447 |
|  |  |  | Interaction | F (1, 20) = 0.01703 | 0.8975 |
| **Supplementary Figure S8D** |  |  |  |  |  |
| CX3CR1 | Two-way ANOVA (sex x genotype) | WT: 6/6  APP/PS1:6/6 | Sex | F (1, 20) = 1.305 | 0.2668 |
|  |  |  | Genotype | F (1, 20) = 1.243 | 0.2781 |
|  |  |  | Interaction | F (1, 20) = 0.003734 | 0.9519 |
| **Supplementary Figure S8E** |  |  |  |  |  |
| C1q | Two-way ANOVA (sex x genotype) | WT: 6/6  APP/PS1:6/6 | Sex | F (1, 20) = 0.1061 | 0.7480 |
|  |  |  | Genotype | F (1, 20) = 2.688 | 0.1168 |
|  |  |  | Interaction | F (1, 20) = 0.6068 | 0.4451 |
| **Supplementary Figure S8F** |  |  |  |  |  |
| TNF-α | Two-way ANOVA (sex x genotype) | WT: 6/6  APP/PS1:6/5 | Sex | F (1, 19) = 0.3472 | 0.5626 |
|  |  |  | Genotype | F (1, 19) = 0.3259 | 0.5748 |
|  |  |  | Interaction | F (1, 19) = 0.8555 | 0.3666 |
| **Supplementary Figure S8G** |  |  |  |  |  |
| IL-1β | Two-way ANOVA (sex x genotype) | WT: 6/6  APP/PS1:6/6 | Sex | F (1, 20) = 3.558 | 0.0739 |
|  |  |  | Genotype | F (1, 20) = 2.387 | 0.1380 |
|  |  |  | Interaction | F (1, 20) = 2.149 | 0.1582 |
| **Supplementary Figure S8H** |  |  |  |  |  |
| NFκB1 | Two-way ANOVA (sex x genotype) | WT: 6/6  APP/PS1:6/6 | Sex | F (1, 20) = 4.672 | 0.0430 |
|  |  |  | Genotype | F (1, 20) = 0.8687 | 0.3624 |
|  |  |  | Interaction | F (1, 20) = 0.4053 | 0.5316 |
| **Supplementary Figure S8I** |  |  |  |  |  |
| Cox-2 | Two-way ANOVA (sex x genotype) | WT: 6/6  APP/PS1:6/6 | Sex | F (1, 20) = 0.1223 | 0.7302 |
|  |  |  | Genotype | F (1, 20) = 0.1103 | 0.7433 |
|  |  |  | Interaction | F (1, 20) = 0.0009570 | 0.9756 |
| **Supplementary Figure S8J** |  |  |  |  |  |
| IL-6 | Two-way ANOVA (sex x genotype) | WT: 6/6  APP/PS1:6/6 | Sex | F (1, 20) = 0.2861 | 0.5986 |
|  |  |  | Genotype | F (1, 20) = 0.3833 | 0.5428 |
|  |  |  | Interaction | F (1, 20) = 0.4307 | 0.5191 |
| **Supplementary Figure S9A** |  |  |  |  |  |
| GFAP | Two-way ANOVA (sex x genotype) | WT: 6/5  APP/PS1:5/5 | Sex | F (1, 17) = 6.824 | 0.0182 |
|  |  |  | Genotype | F (1, 17) = 0.04910 | 0.8273 |
|  |  |  | Interaction | F (1, 17) = 0.03637 | 0.8510 |
| **Supplementary Figure S9B** |  |  |  |  |  |
| iNOS | Two-way ANOVA (sex x genotype) | WT: 6/6  APP/PS1:5/6 | Sex | F (1, 19) = 0.7265 | 0.4046 |
|  |  |  | Genotype | F (1, 19) = 5.110 | 0.0357 |
|  |  |  | Interaction | F (1, 19) = 0.1472 | 0.7054 |
| **Supplementary Figure S9C** |  |  |  |  |  |
| C3 | Two-way ANOVA (sex x genotype) | WT: 5/6  APP/PS1:5/6 | Sex | F (1, 18) = 0.9135 | 0.3518 |
|  |  |  | Genotype | F (1, 18) = 0.08930 | 0.7685 |
|  |  |  | Interaction | F (1, 18) = 2.697 | 0.1179 |
| **Supplementary Figure S9D** |  |  |  |  |  |
| TGFβ | Two-way ANOVA (sex x genotype) | WT: 6/6  APP/PS1:5/6 | Sex | F (1, 19) = 9.217 | 0.0068 |
|  |  |  | Genotype | F (1, 19) = 0.4039 | 0.5327 |
|  |  |  | Interaction | F (1, 19) = 0.8894 | 0.3575 |
| **Supplementary Figure S9E** |  |  |  |  |  |
| S100a10 | Two-way ANOVA (sex x genotype) | WT: 6/6  APP/PS1:5/6 | Sex | F (1, 19) = 1.652 | 0.2141 |
|  |  |  | Genotype | F (1, 19) = 0.6289 | 0.4375 |
|  |  |  | Interaction | F (1, 19) = 0.5696 | 0.4597 |
| **Supplementary Figure S9F** |  |  |  |  |  |
| CD68 | Two-way ANOVA (sex x genotype) | WT: 6/5  APP/PS1:5/6 | Sex | F (1, 18) = 0.5184 | 0.4808 |
|  |  |  | Genotype | F (1, 18) = 0.2794 | 0.6036 |
|  |  |  | Interaction | F (1, 18) = 3.804 | 0.0669 |
| **Supplementary Figure S9G** |  |  |  |  |  |
| CX3CR1 | Two-way ANOVA (sex x genotype) | WT: 6/6  APP/PS1:5/6 | Sex | F (1, 19) = 3.542 | 0.0752 |
|  |  |  | Genotype | F (1, 19) = 0.04307 | 0.8378 |
|  |  |  | Interaction | F (1, 19) = 0.02542 | 0.8750 |
| **Supplementary Figure S9H** |  |  |  |  |  |
| C1q | Two-way ANOVA (sex x genotype) | WT: 6/6  APP/PS1:5/6 | Sex | F (1, 19) = 4.753 | 0.0420 |
|  |  |  | Genotype | F (1, 19) = 0.007873 | 0.9302 |
|  |  |  | Interaction | F (1, 19) = 0.02987 | 0.8646 |
| **Supplementary Figure S9I** |  |  |  |  |  |
| TNF-α | Two-way ANOVA (sex x genotype) | WT: 5/6  APP/PS1:5/6 | Sex | F (1, 18) = 0.5472 | 0.4690 |
|  |  |  | Genotype | F (1, 18) = 0.03609 | 0.8514 |
|  |  |  | Interaction | F (1, 18) = 0.7093 | 0.4107 |
| **Supplementary Figure S9J** |  |  |  |  |  |
| IL-1β | Two-way ANOVA (sex x genotype) | WT: 6/6  APP/PS1:5/6 | Sex | F (1, 19) = 2.274 | 0.1480 |
|  |  |  | Genotype | F (1, 19) = 0.5227 | 0.4785 |
|  |  |  | Interaction | F (1, 19) = 4.080 | 0.0577 |
| **Supplementary Figure S9K** |  |  |  |  |  |
| BDNF | Two-way ANOVA (sex x genotype) | WT: 6/6  APP/PS1:5/6 | Sex | F (1, 19) = 9.638 | 0.0058 |
|  |  |  | Genotype | F (1, 19) = 1.239 | 0.2795 |
|  |  |  | Interaction | F (1, 19) = 1.693 | 0.2088 |
| **Supplementary Figure S9L** |  |  |  |  |  |
| NFκB1 | Two-way ANOVA (sex x genotype) | WT: 6/6  APP/PS1:5/6 | Sex | F (1, 19) = 0.6953 | 0.4147 |
|  |  |  | Genotype | F (1, 19) = 0.6004 | 0.4480 |
|  |  |  | Interaction | F (1, 19) = 0.9657 | 0.3381 |
| **Supplementary Figure S9M** |  |  |  |  |  |
| NFκB2 | Two-way ANOVA (sex x genotype) | WT: 6/6  APP/PS1:5/6 | Sex | F (1, 19) = 1.743 | 0.2025 |
|  |  |  | Genotype | F (1, 19) = 0.003536 | 0.9532 |
|  |  |  | Interaction | F (1, 19) = 0.4656 | 0.5032 |
| **Supplementary Figure S9N** |  |  |  |  |  |
| Cox-2 | Two-way ANOVA (sex x genotype) | WT: 6/6  APP/PS1:5/6 | Sex | F (1, 19) = 4.573 | 0.0457 |
|  |  |  | Genotype | F (1, 19) = 0.5087 | 0.4844 |
|  |  |  | Interaction | F (1, 19) = 0.3897 | 0.5399 |
| **Supplementary Figure S9O** |  |  |  |  |  |
| IL-6 | Two-way ANOVA (sex x genotype) | WT: 6/6  APP/PS1:4/6 | Sex | F (1, 17) = 0.6968 | 0.4154 |
|  |  |  | Genotype | F (1, 17) = 0.2036 | 0.6575 |
|  |  |  | Interaction | F (1, 17) = 3.318 | 0.0862 |
| **Supplementary Figure S11A** |  |  |  |  |  |
| Sdhb | Two-way ANOVA (sex x genotype) | WT: 6/6  APP/PS1:6/6 | Sex | F (1, 20) = 0.5768 | 0.4564 |
|  |  |  | Genotype | F (1, 20) = 3.452 | 0.0779 |
|  |  |  | Interaction | F (1, 20) = 0.7301 | 0.4030 |
| **Supplementary Figure S11B** |  |  |  |  |  |
| Uqcrc1 | Two-way ANOVA (sex x genotype) | WT: 6/6  APP/PS1:6/6 | Sex | F (1, 20) = 0.1584 | 0.6948 |
|  |  |  | Genotype | F (1, 20) = 3.707 | 0.0685 |
|  |  |  | Interaction | F (1, 20) = 0.3296 | 0.5723 |
| **Supplementary Figure S11C** |  |  |  |  |  |
| Cox5b | Two-way ANOVA (sex x genotype) | WT: 6/6  APP/PS1:6/6 | Sex | F (1, 20) = 4.389 | 0.0491 |
|  |  |  | Genotype | F (1, 20) = 2.766 | 0.1119 |
|  |  |  | Interaction | F (1, 20) = 2.144 | 0.1586 |
| **Supplementary Figure S12A** |  |  |  |  |  |
| GFAP^EE^ | Two-way ANOVA (sex x genotype) | WT: 7/7  APP/PS1:7/7 | Sex | F (1, 24) = 1.880 | 0.1830 |
|  |  |  | Genotype | F (1, 24) = 1.890 | 0.1819 |
|  |  |  | Interaction | F (1, 24) = 0.03228 | 0.8589 |
| **Supplementary Figure S12B** |  |  |  |  |  |
| C3^EE^ | Two-way ANOVA (sex x genotype) | WT: 7/7  APP/PS1:7/7 | Sex | F (1, 24) = 1.384 | 0.2509 |
|  |  |  | Genotype | F (1, 24) = 0.2342 | 0.6328 |
|  |  |  | Interaction | F (1, 24) = 0.05736 | 0.8128 |
| **Supplementary Figure S12C** |  |  |  |  |  |
| NFκB2^EE^ | Two-way ANOVA (sex x genotype) | WT: 7/7  APP/PS1:7/7 | Sex | F (1, 24) = 0.3087 | 0.5836 |
|  |  |  | Genotype | F (1, 24) = 3.633 | 0.0687 |
|  |  |  | Interaction | F (1, 24) = 4.095 | 0.0543 |
| **Supplementary Figure S12D** |  |  |  |  |  |
| BDNF^EE^ | Two-way ANOVA (sex x genotype) | WT: 7/7  APP/PS1:7/7 | Sex | F (1, 24) = 15.17 | 0.0007 |
|  |  |  | Genotype | F (1, 24) = 3.839 | 0.0618 |
|  |  |  | Interaction | F (1, 24) = 0.1791 | 0.6759 |
| **Supplementary Figure S12E** |  |  |  |  |  |
| IL-1β^EE^ | Two-way ANOVA (sex x genotype) | WT: 5/7  APP/PS1:6/7 | Sex | F (1, 21) = 12.24 | 0.0021 |
|  |  |  | Genotype | F (1, 21) = 2.659 | 0.1178 |
|  |  |  | Interaction | F (1, 21) = 0.01486 | 0.9041 |
| **Supplementary Figure S12F** |  |  |  |  |  |
| MCT2^EE^ | Two-way ANOVA (sex x genotype) | WT: 7/7  APP/PS1:7/7 | Sex | F (1, 24) = 0.3014 | 0.5881 |
|  |  |  | Genotype | F (1, 24) = 3.780 | 0.0637 |
|  |  |  | Interaction | F (1, 24) = 2.431 | 0.1321 |
| **Supplementary Figure S12G** |  |  |  |  |  |
| Ndufs8^EE^ | Two-way ANOVA (sex x genotype) | WT: 7/7  APP/PS1:7/7 | Sex | F (1, 24) = 1.562 | 0.2235 |
|  |  |  | Genotype | F (1, 24) = 0.7723 | 0.3882 |
|  |  |  | Interaction | F (1, 24) = 0.2200 | 0.6433 |
| **Supplementary Figure S12H** |  |  |  |  |  |
| Atp5a1^EE^ | Two-way ANOVA (sex x genotype) | WT: 7/7  APP/PS1:7/7 | Sex | F (1, 24) = 0.01638 | 0.8992 |
|  |  |  | Genotype | F (1, 24) = 0.02016 | 0.8883 |
|  |  |  | Interaction | F (1, 24) = 1.657 | 0.2103 |
| **Supplementary Figure S13A** |  |  |  |  |  |
| GFAP-RN | Two-way ANOVA (sex x genotype) | WT: 7/7  APP/PS1:7/7 | Sex | F (1, 24) = 0.2411 | 0.6278 |
|  |  |  | Genotype | F (1, 24) = 0.3811 | 0.5428 |
|  |  |  | Interaction | F (1, 24) = 0.04455 | 0.8346 |
| **Supplementary Figure S13B** |  |  |  |  |  |
| Iba1-RN | Two-way ANOVA (sex x genotype) | WT: 6/6  APP/PS1:6/6 | Sex | F (1, 20) = 0.8376 | 0.3710 |
|  |  |  | Genotype | F (1, 20) = 0.008394 | 0.9279 |
|  |  |  | Interaction | F (1, 20) = 0.1145 | 0.7386 |
| **Supplementary Figure S14** |  |  |  |  |  |
| LC astrocyte structure | Mann-Whitney U test | Female WT: 6  Female APP/PS1:6  (2-3 astrocytes/animal) | - | U=1137  Median (WT)=5.125  Median (APP/PS1) =5.500 | 0.9114 |
| **Supplementary Figure S15B** |  |  |  |  |  |
| LC-TH | Two-way ANOVA (sex x genotype) | WT: 7/7  APP/PS1:7/7 | Sex | F (1, 24) = 6.027 | 0.0217 |
|  |  |  | Genotype | F (1, 24) = 0.02186 | 0.8837 |
|  |  |  | Interaction | F (1, 24) = 3.990 | 0.0572 |
| **Supplementary Figure S15C** |  |  |  |  |  |
| LC-soma volume | Two-way ANOVA (sex x genotype) | WT: 5/5  APP/PS1:5/5 | Sex | F (1, 16) = 0.4415 | 0.5159 |
|  |  |  | Genotype | F (1, 16) = 0.1918 | 0.6673 |
|  |  |  | Interaction | F (1, 16) = 0.4224 | 0.5250 |
| **Supplementary Figure S15D** |  |  |  |  |  |
| LC-TH^EE^ | Two-way ANOVA (sex x genotype) | WT: 6/5  APP/PS1:6/6 | Sex | F (1, 19) = 4.009 | 0.0598 |
|  |  |  | Genotype | F (1, 19) = 0.04295 | 0.8380 |
|  |  |  | Interaction | F (1, 19) = 0.9872 | 0.3329 |
| **Supplementary Figure S15F** |  |  |  |  |  |
| LC-NET^EE^  Soma | Two-way ANOVA (sex x genotype) | WT: 3/3  APP/PS1:3/3 | Sex | F (1, 8) =0.4053 | 0.5421 |
|  |  |  | Genotype | F (1, 8) = 0.5544 | 0.4778 |
|  |  |  | Interaction | F (1, 8) = 0.01846 | 0.8953 |
| LC-NET^EE^  Dendritic region | Two-way ANOVA (sex x genotype | WT: 3/3  APP/PS1:3/3 | Sex | F (1, 8) =0.8349 | 0.3876 |
|  |  |  | Genotype | F (1, 8) = 1.007 | 0.3449 |
|  |  |  | Interaction | F (1, 8) = 0.2061 | 0.6619 |
| LC-NET^EE^  Somatodendritic region | Two-way ANOVA (sex x genotype) | WT: 3/3  APP/PS1:3/3 | Sex | F (1, 8) =0.05254 | 0.8244 |
|  |  |  | Genotype | F (1, 8) = 0.4260 | 0.5322 |
|  |  |  | Interaction | F (1, 8) = 0.05021 | 0.8283 |
| **Supplementary Figure S16B** |  |  |  |  |  |
| LC-Total α2AR  Soma | Two-way ANOVA (sex x genotype) | WT: 3/3  APP/PS1:3/3 | Sex | F (1, 8) =1.055 | 0.3344 |
|  |  |  | Genotype | F (1, 8) = 0.07905 | 0.7857 |
|  |  |  | Interaction | F (1, 8) = 0.08015 | 0.7843 |
| LC-Total α2AR  Dendritic region | Two-way ANOVA (sex x genotype) | WT: 3/3  APP/PS1:3/3 | Sex | F (1, 8) =0.02181 | 0.8863 |
|  |  |  | Genotype | F (1, 8) = 0.07696 | 0.7885 |
|  |  |  | Interaction | F (1, 8) = 0.009689 | 0.9240 |
| LC-Total α2AR  Somatodendritic region | Two-way ANOVA (sex x genotype) | WT: 3/3  APP/PS1:3/3 | Sex | F (1, 8) =0.3271 | 0.5831 |
|  |  |  | Genotype | F (1, 8) = 0.0003446 | 0.9856 |
|  |  |  | Interaction | F (1, 8) = 0.03588 | 0.8545 |
| **Supplementary Figure S16C** |  |  |  |  |  |
| LC-Total α2AR^EE^  Soma | Two-way ANOVA (sex x genotype) | WT: 3/3  APP/PS1:3/3 | Sex | F (1, 8) =0.3863 | 0.5515 |
|  |  |  | Genotype | F (1, 8) = 0.1282 | 0.7296 |
|  |  |  | Interaction | F (1, 8) = 0.01203 | 0.9154 |
| LC-Total α2AR^EE^  Dendritic region | Two-way ANOVA (sex x genotype) | WT: 3/3  APP/PS1:3/3 | Sex | F (1, 8) =0.001891 | 0.9664 |
|  |  |  | Genotype | F (1, 8) = 1.596e-005 | 0.9969 |
|  |  |  | Interaction | F (1, 8) = 0.1234 | 0.7344 |
| LC-Total α2AR^EE^  Somatodendritic region | Two-way ANOVA (sex x genotype) | WT: 3/3  APP/PS1:3/3 | Sex | F (1, 8) =0.1119 | 0.7466 |
|  |  |  | Genotype | F (1, 8) = 0.03308 | 0.8602 |
|  |  |  | Interaction | F (1, 8) = 0.05520 | 0.8201 |
| **Supplementary Figure S16D** |  |  |  |  |  |
| LC-Astrocytic α2AR^EE^  Soma | Two-way ANOVA (sex x genotype) | WT: 3/3  APP/PS1:3/3  (29-40 astrocytes/group) | Sex | F (1, 134) =2.113 | 0.1484 |
|  |  |  | Genotype | F (1, 134) =2.132 | 0.1466 |
|  |  |  | Interaction | F (1, 134) =3.232 | 0.0745 |
| LC-Astrocytic α2AR^EE^  Dendritic region | Two-way ANOVA (sex x genotype) | WT: 3/3  APP/PS1:3/3  (28-40 astrocytes/group) | Sex | F (1, 132) = 1.186 | 0.2782 |
|  |  |  | Genotype | F (1, 132) = 3.602 | 0.0599 |
|  |  |  | Interaction | F (1, 132) = 4.178 | 0.0429 |
| LC-Astrocytic α2AR^EE^  Somatodendritic region | Two-way ANOVA (sex x genotype) | WT: 3/3  APP/PS1:3/3  (67-70 astrocytes/group) | Sex | F (1, 270) = 0.4807 | 0.4887 |
|  |  |  | Genotype | F (1, 270) = 0.001020 | 0.9745 |
|  |  |  | Interaction | F (1, 270) = 4.941 | 0.0271 |

Note: All data are represented as Mean ± SEM. All two-way ANOVA tests were followed by Tukey-Kramer’s multiple comparisons test.

Abbreviations: α2AR, Alpha 2A adrenergic receptor; Aβ42, amyloid-β 42; Atp5a1, ATP synthase F1 subunit α; BACE1, β-Site APP-cleaving enzyme-1; BDNF, brain-derived neurotrophic factor; C1q, complement component 1q; C3, complement component 3; CD68, cluster of differentiation 68; Cox-2, cyclooxygenase-2; Cox5b, cytochrome c oxidase subunit 5b; Cr+PCr, creatine+phosphocreatine; CX3CR1, CX3C chemokine receptor 1; DBH, dopamine-β-hydroxylase; DMTg, dorsomedial tegmental nucleus; EE, environmental enrichment; GFAP, glial fibrillary acidic protein; Gln, glutamine; Glu, glutamate; Glx, glutamine/glutamate; GPC+PCh, glycerophosphocholine+phosphocholine; GSH, glutathione; Iba1, ionized calcium-binding adaptor molecule 1; iNOS, inducible nitric oxide synthase; IL-1β, interleukin-1 beta; IL-6, interleukin-6; LC, locus coeruleus; MCT2, monocarboxylate transporter 2; mI, myo-inositol; NAA, N-acetyl aspartate; NAA+NAAG, N-Acetylaspartylglutamate; Ndufs8, NADH dehydrogenase iron-sulfur protein 8; NET, norepinephrine transporter; NFκB1, nuclear factor-kappa-B p105/p50 subunit; NFκB2, nuclear factor-kappa-B p100/p52 subunit; pR, pontine raphe; RN, red nucleus; S100a10, S100 calcium binding protein a10; Sdhb, succinate dehydrogenase subunit B; SEM, standard error of mean; SN, substantia nigra; SOX9, SRY-box transcription factor 9; TGFβ, transforming growth factor beta; TH, tyrosine hydroxylase; TNF-α, tumor necrosis factor-alpha; Uqcrc1, ubiquinol-cytochrome-c reductase core protein 1; VTA, ventral tegmental area; WT, wild-type.
