# Supplementary figures and images for: Female‐biased astrocytic priming shapes early locus coeruleus vulnerability in an Aβ oligomer milieu
Source: Alzheimers Dement. 2026 Feb 6;22(2):e71168. doi: 10.1002/alz.71168 (PMC12877963; doi:10.1002/alz.71168)

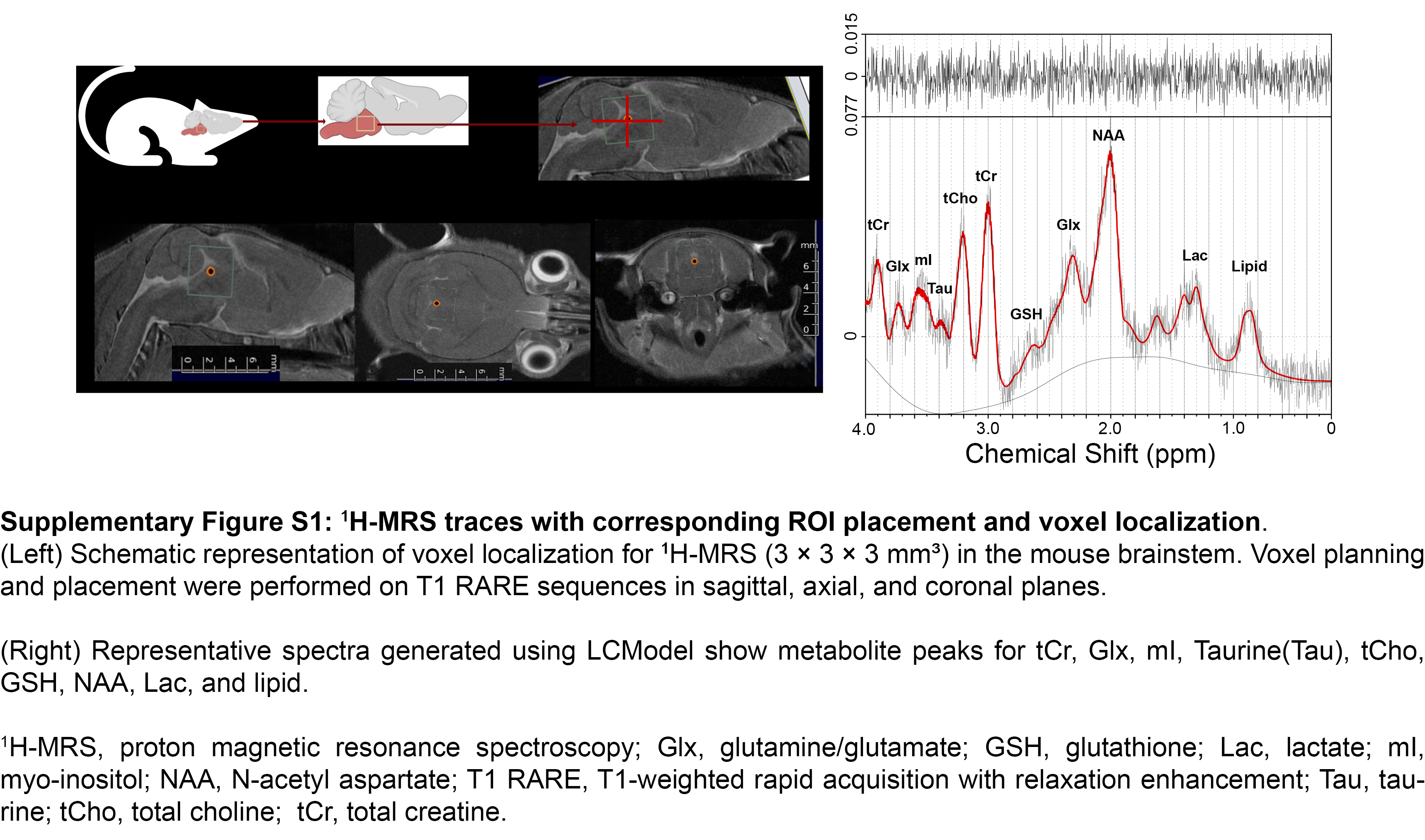

Supplement: Supplementary file 4 — Supporting Information [file ALZ-22-e71168-s011.tif]

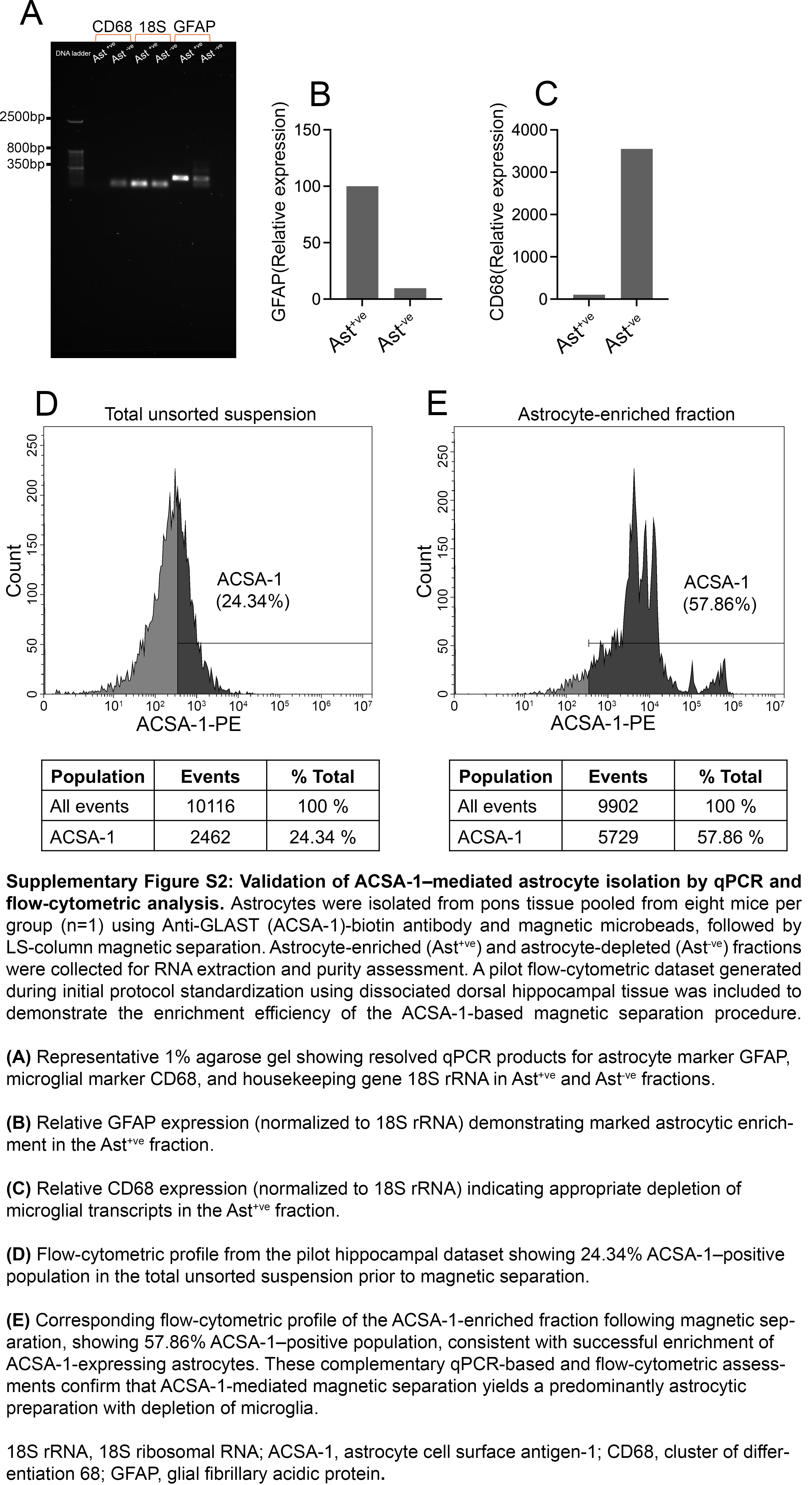

Supplement: Supplementary file 5 — Supporting Information [file ALZ-22-e71168-s002.tif]

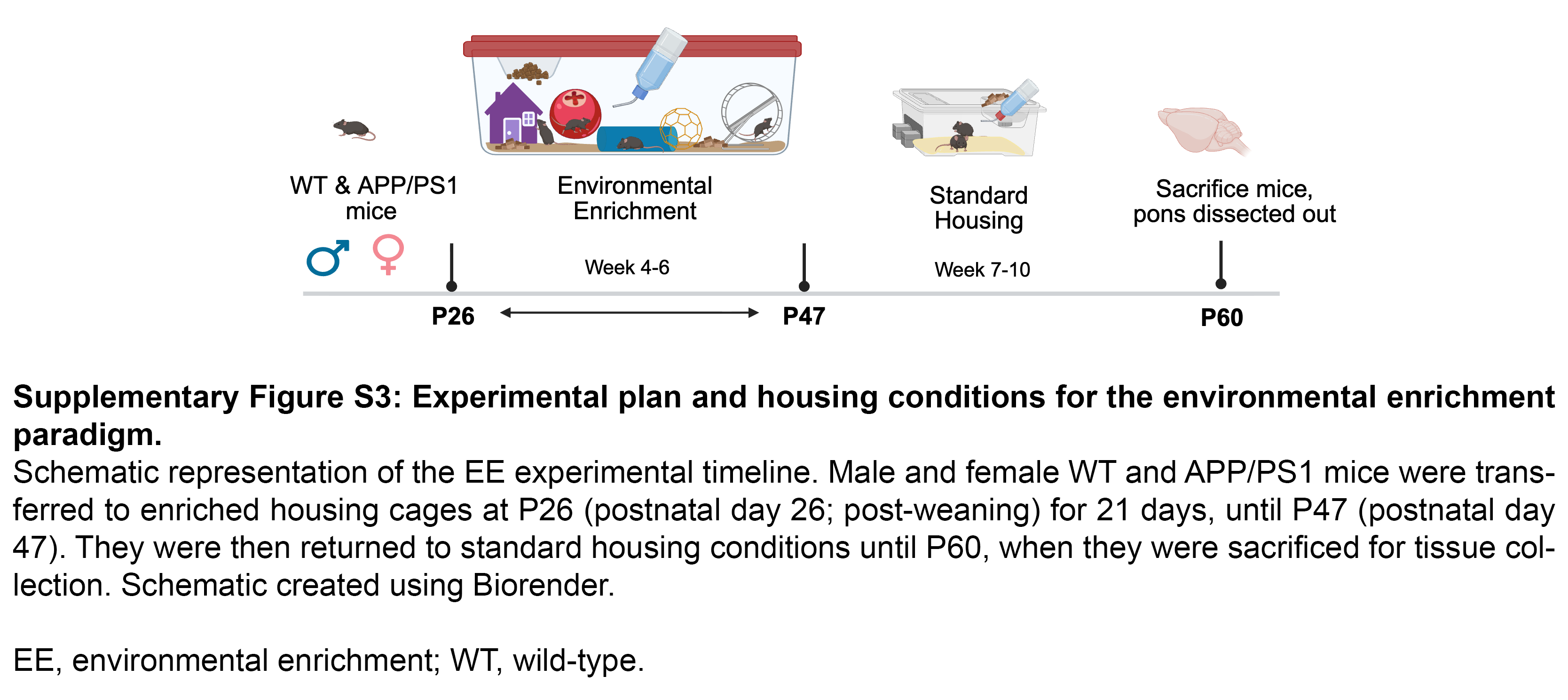

Supplement: Supplementary file 6 — Supporting Information [file ALZ-22-e71168-s017.tif]

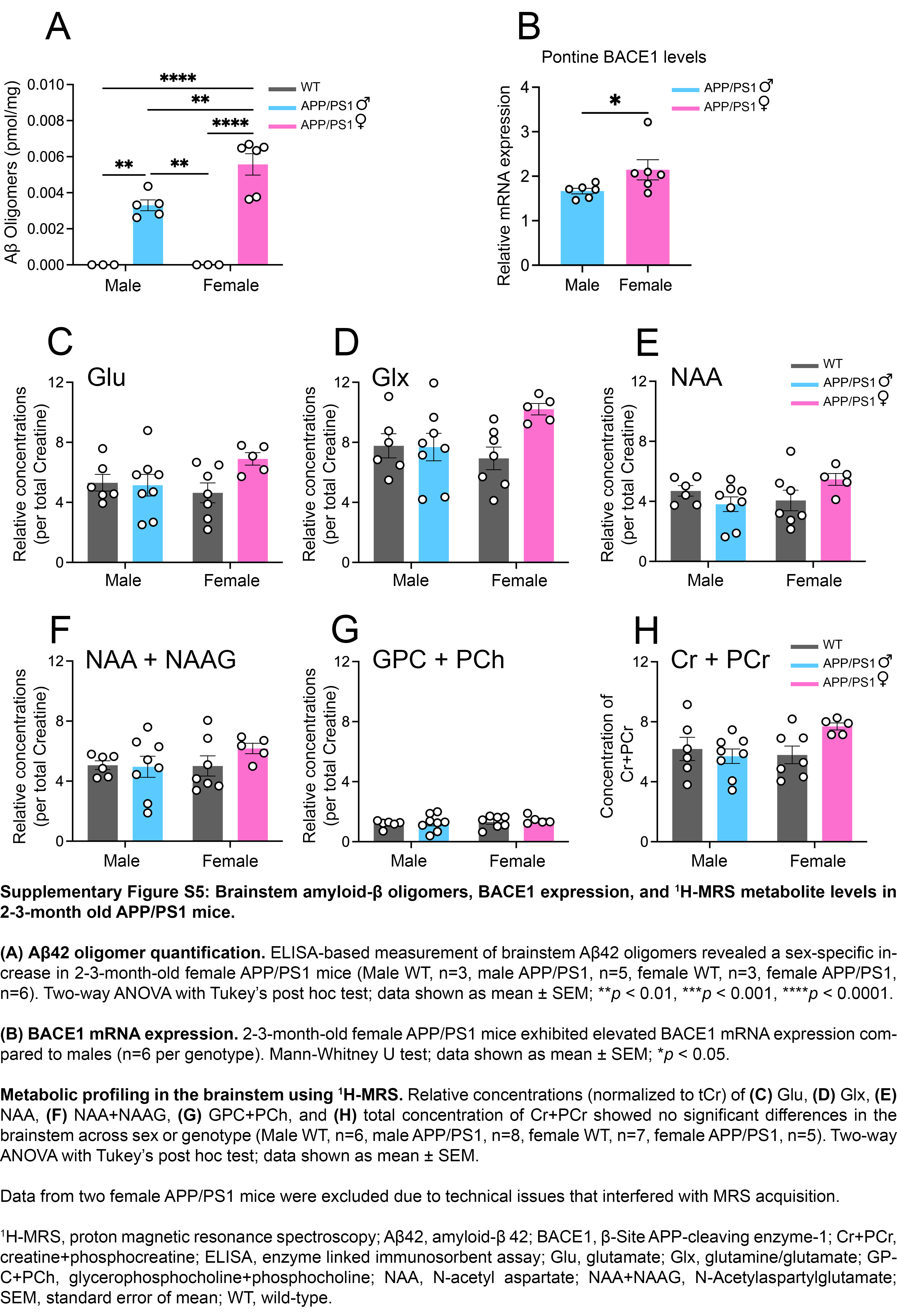

Supplement: Supplementary file 8 — Supporting Information [file ALZ-22-e71168-s019.tif]

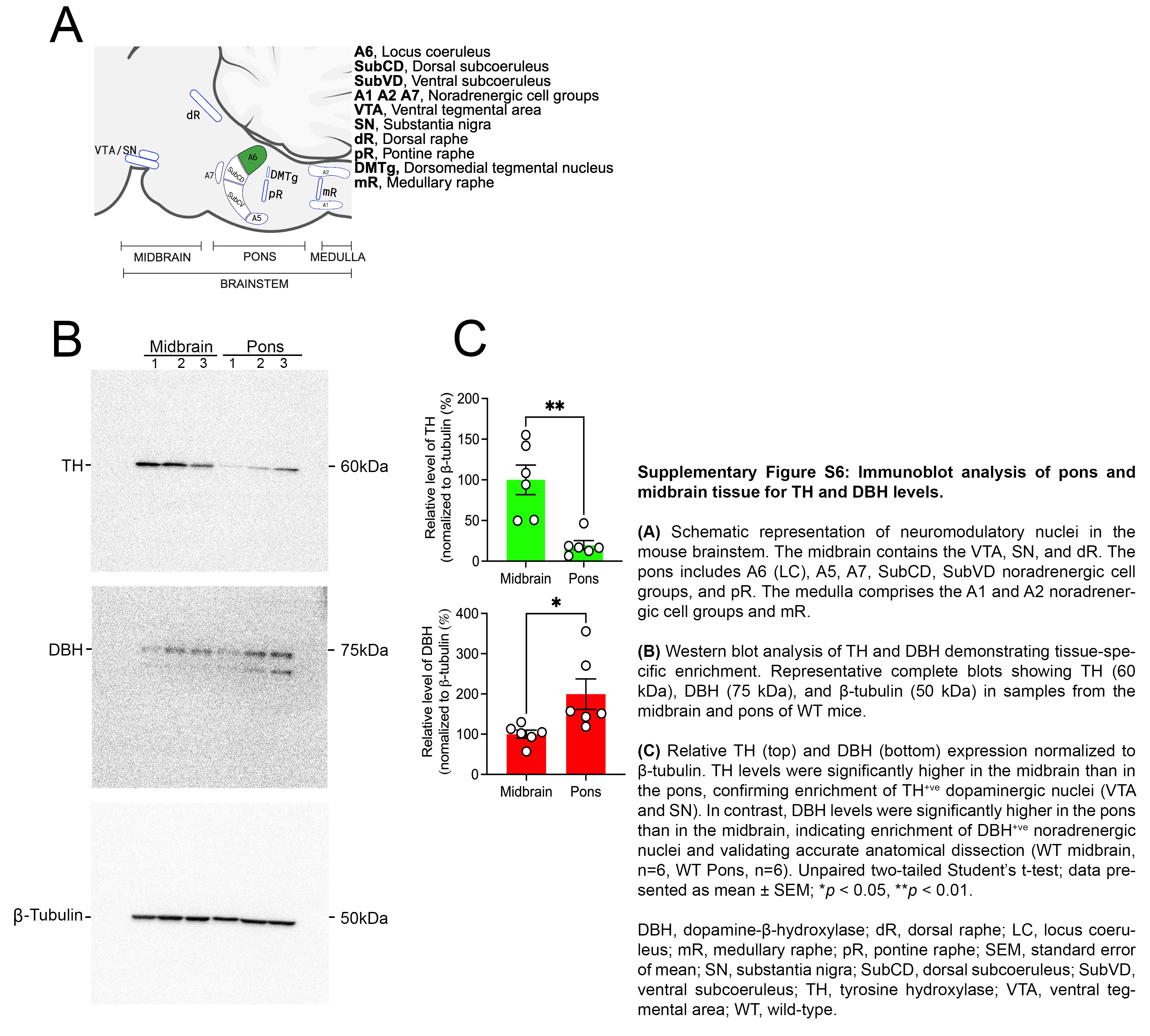

Supplement: Supplementary file 9 — Supporting Information [file ALZ-22-e71168-s010.tif]

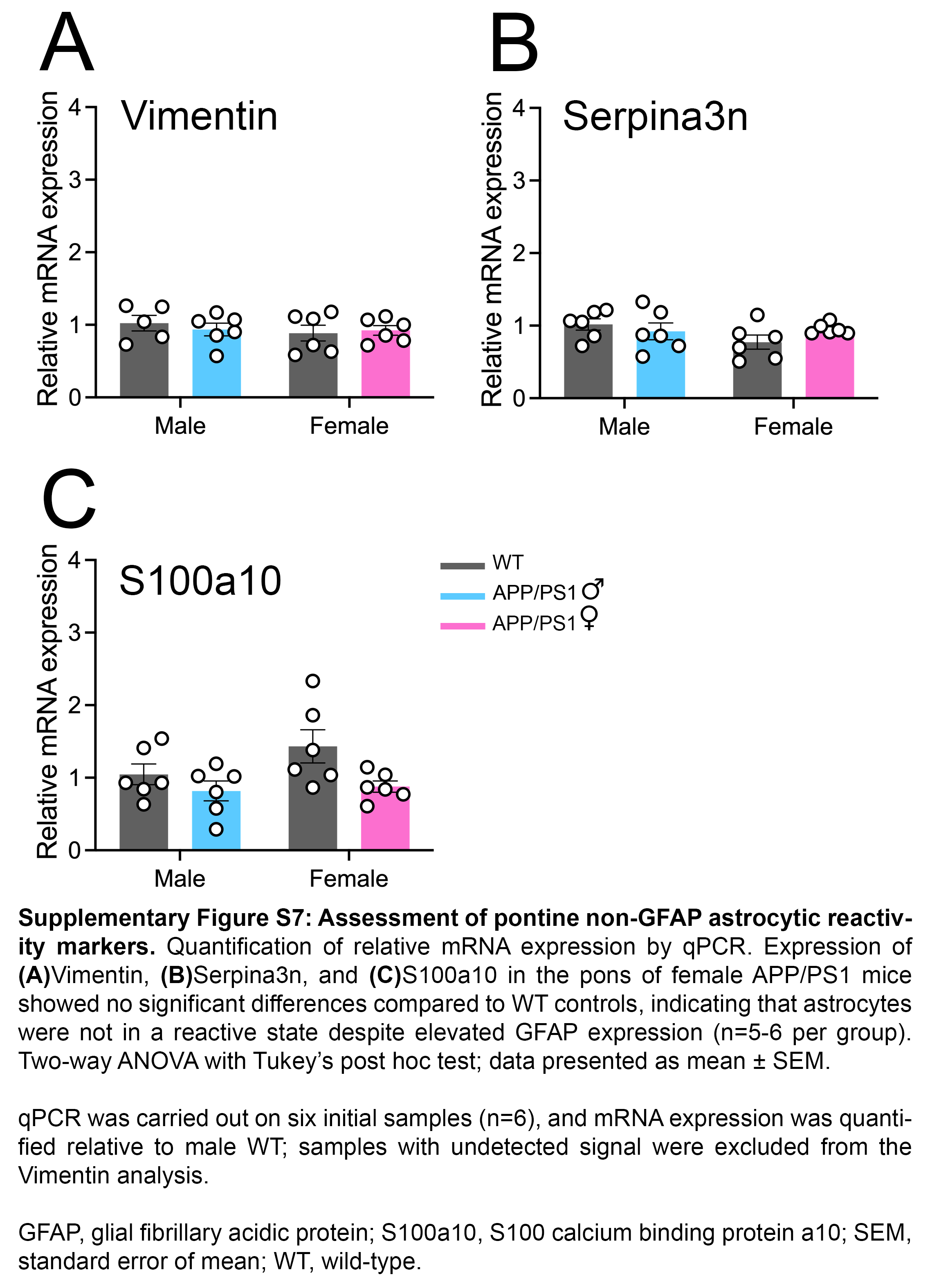

Supplement: Supplementary file 10 — Supporting Information [file ALZ-22-e71168-s009.tif]

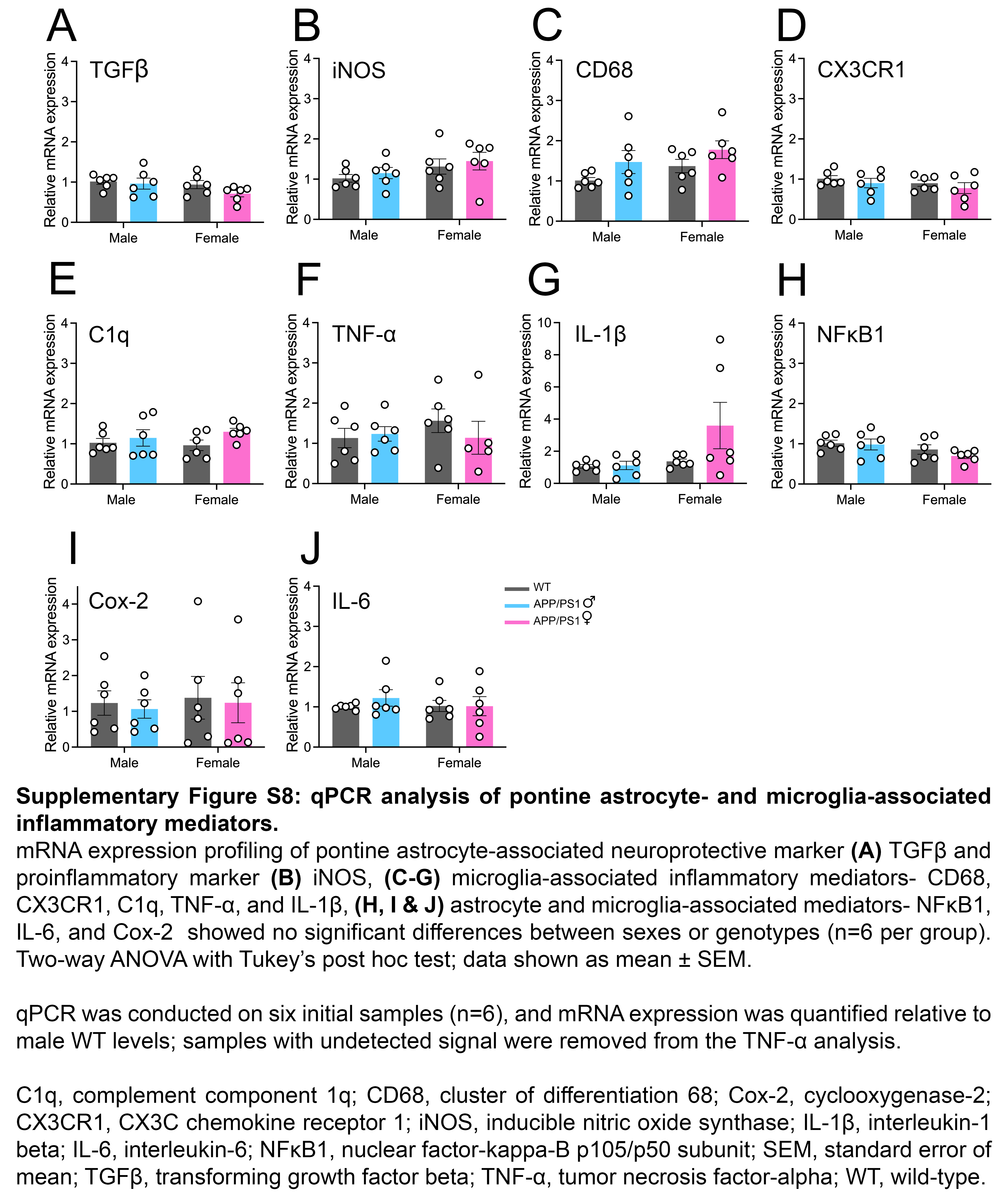

Supplement: Supplementary file 11 — Supporting Information [file ALZ-22-e71168-s007.tif]

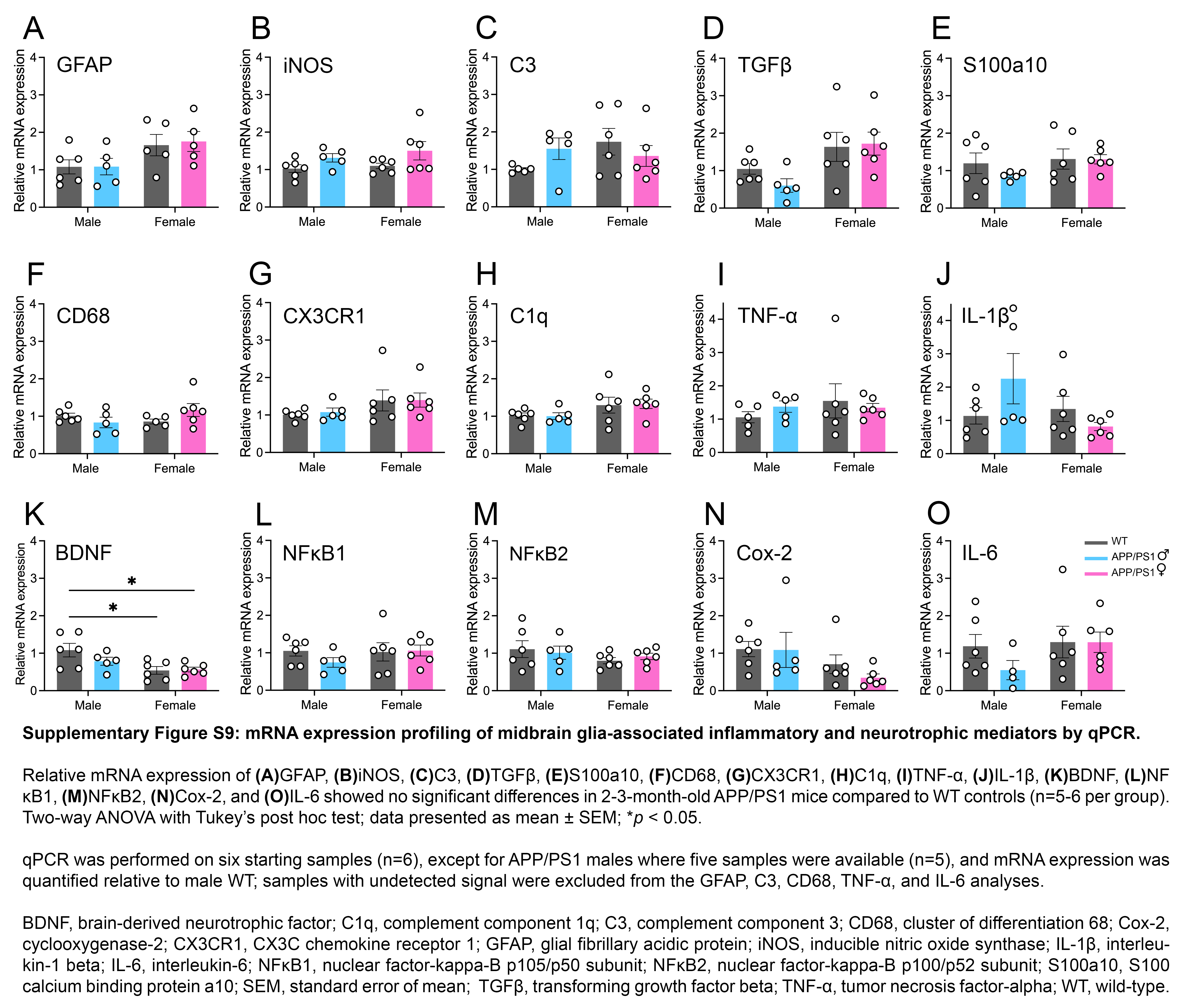

Supplement: Supplementary file 12 — Supporting Information [file ALZ-22-e71168-s013.tif]

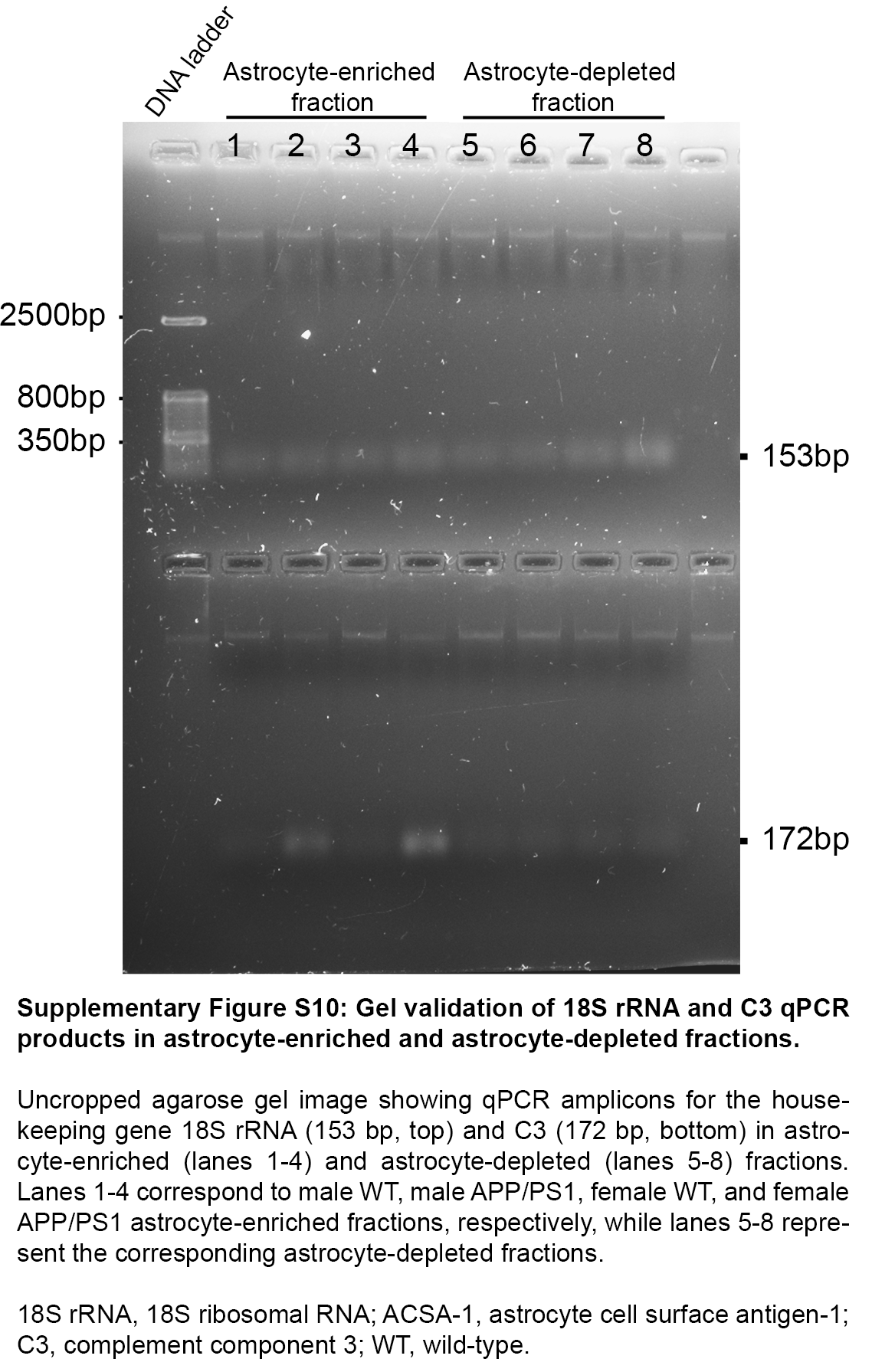

Supplement: Supplementary file 13 — Supporting Information [file ALZ-22-e71168-s008.tif]

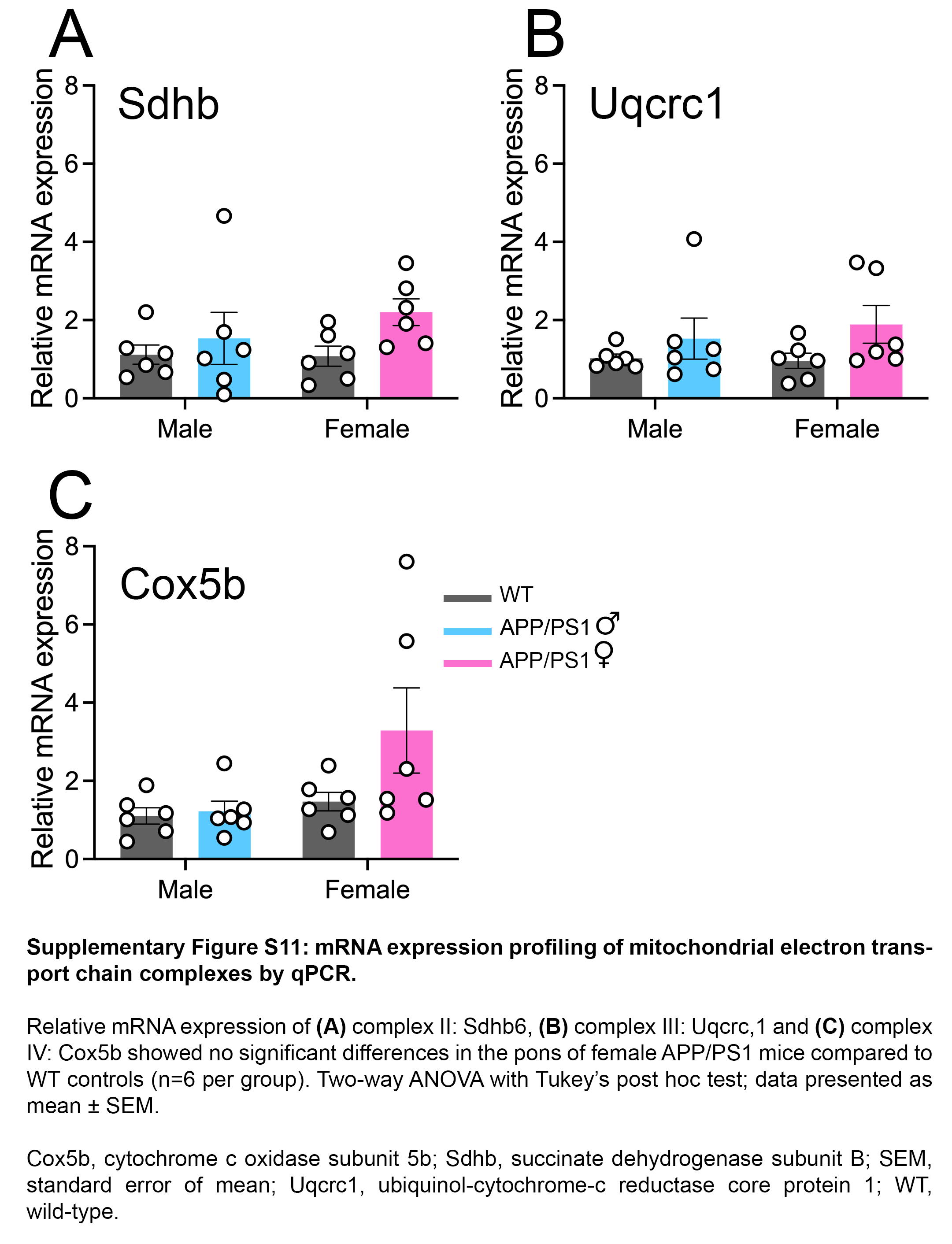

Supplement: Supplementary file 14 — Supporting Information [file ALZ-22-e71168-s018.tif]

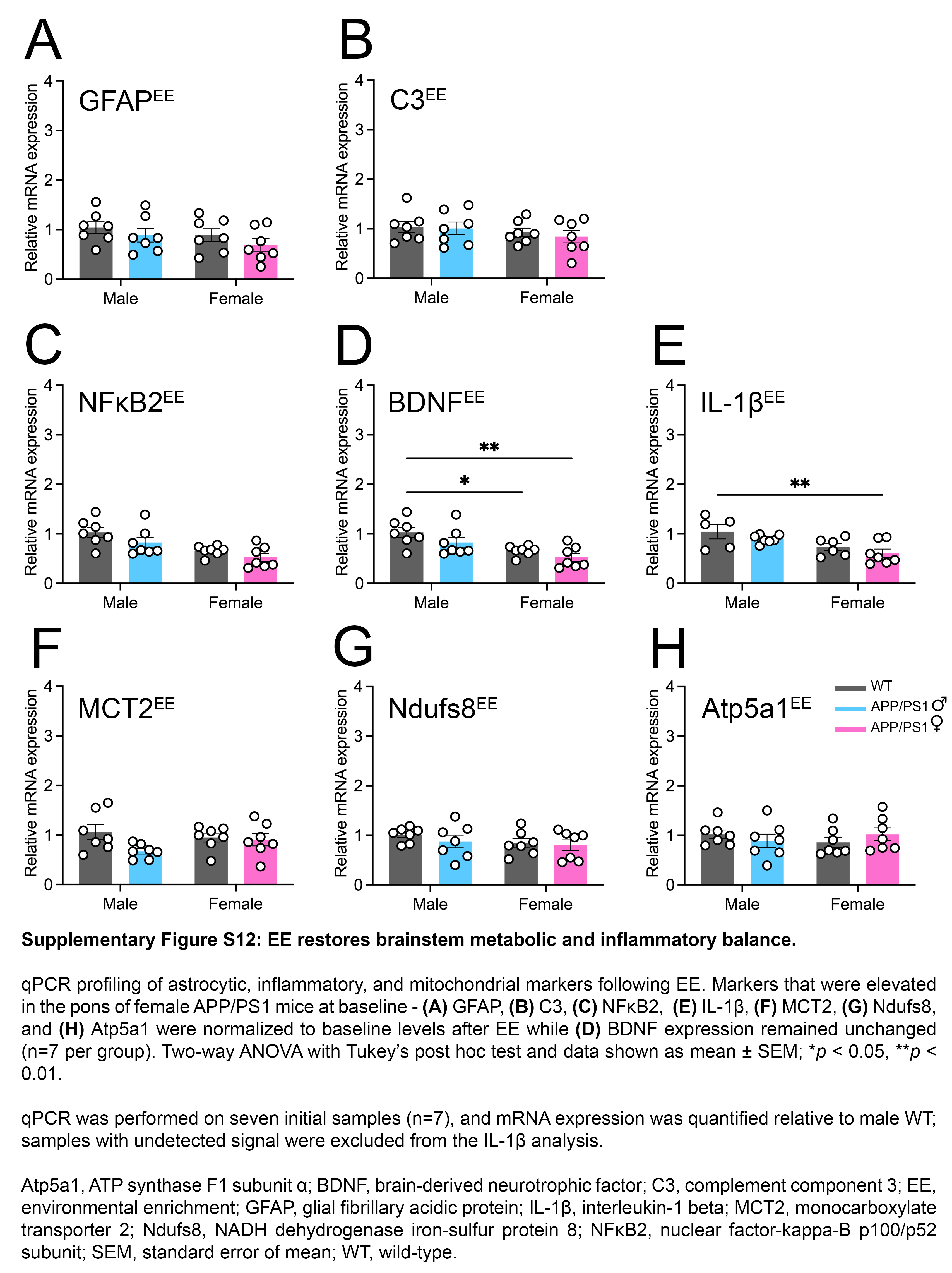

Supplement: Supplementary file 15 — Supporting Information [file ALZ-22-e71168-s012.tif]

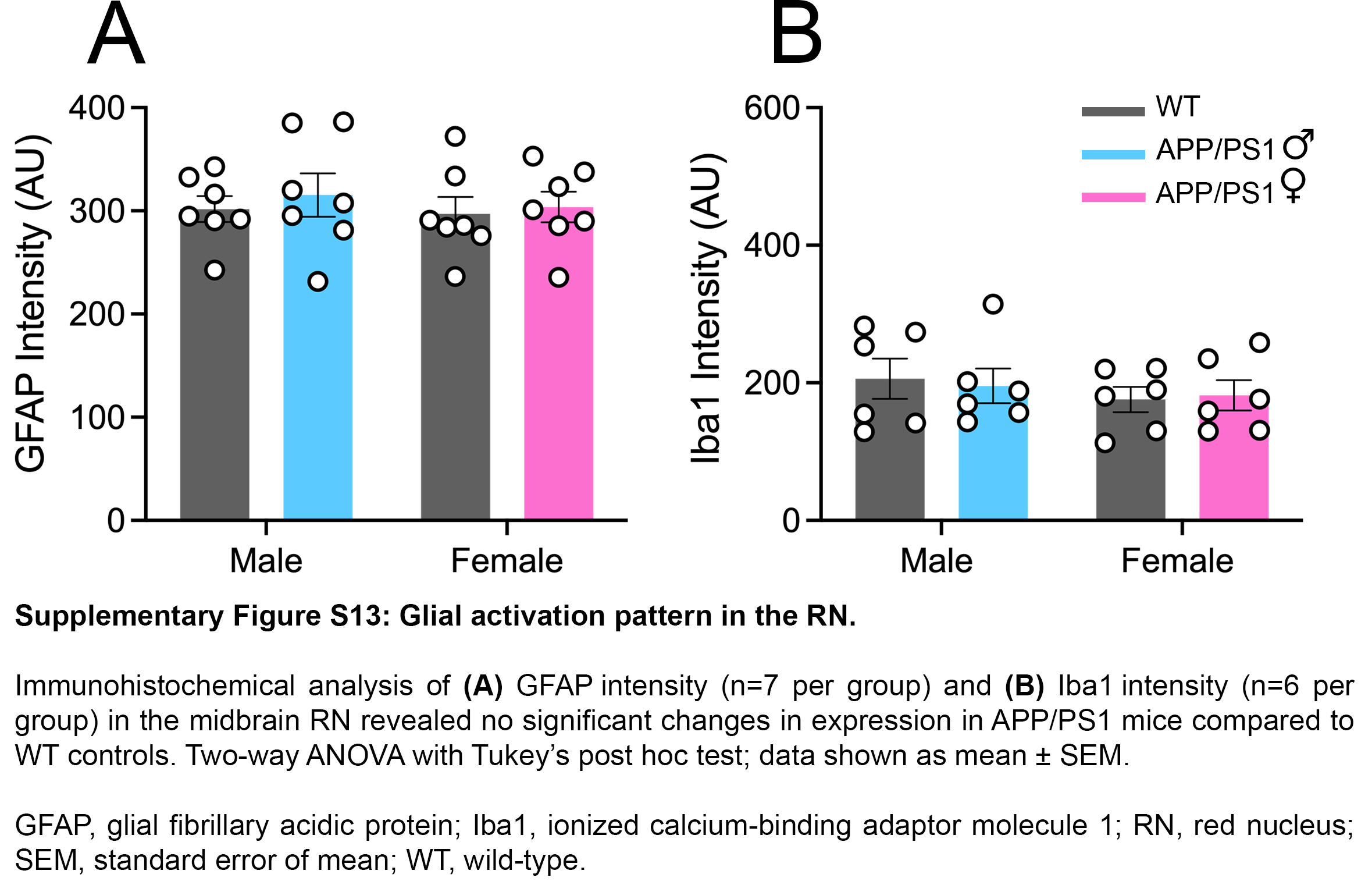

Supplement: Supplementary file 16 — Supporting Information [file ALZ-22-e71168-s020.tif]

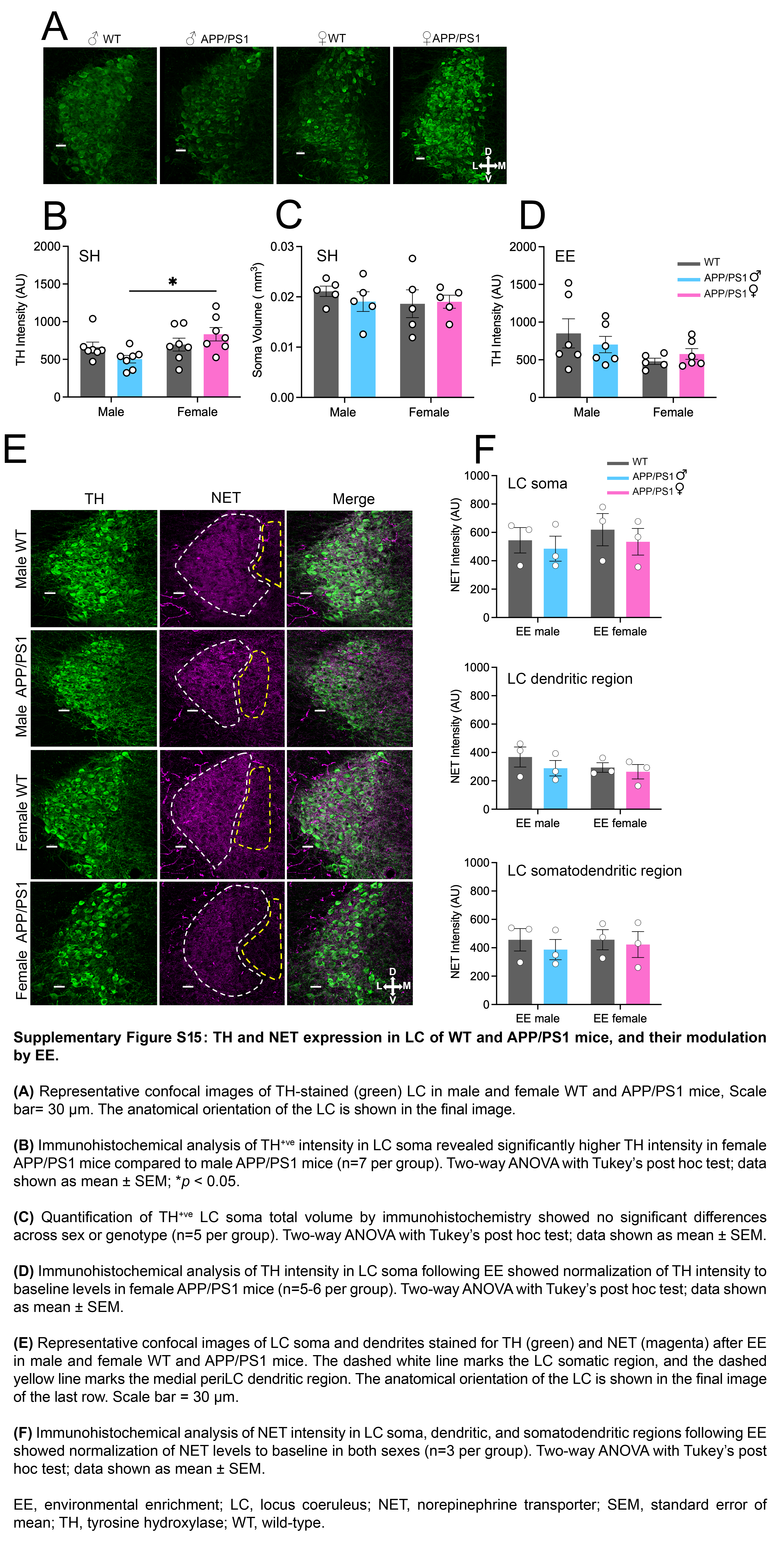

Supplement: Supplementary file 18 — Supporting Information [file ALZ-22-e71168-s006.tif]

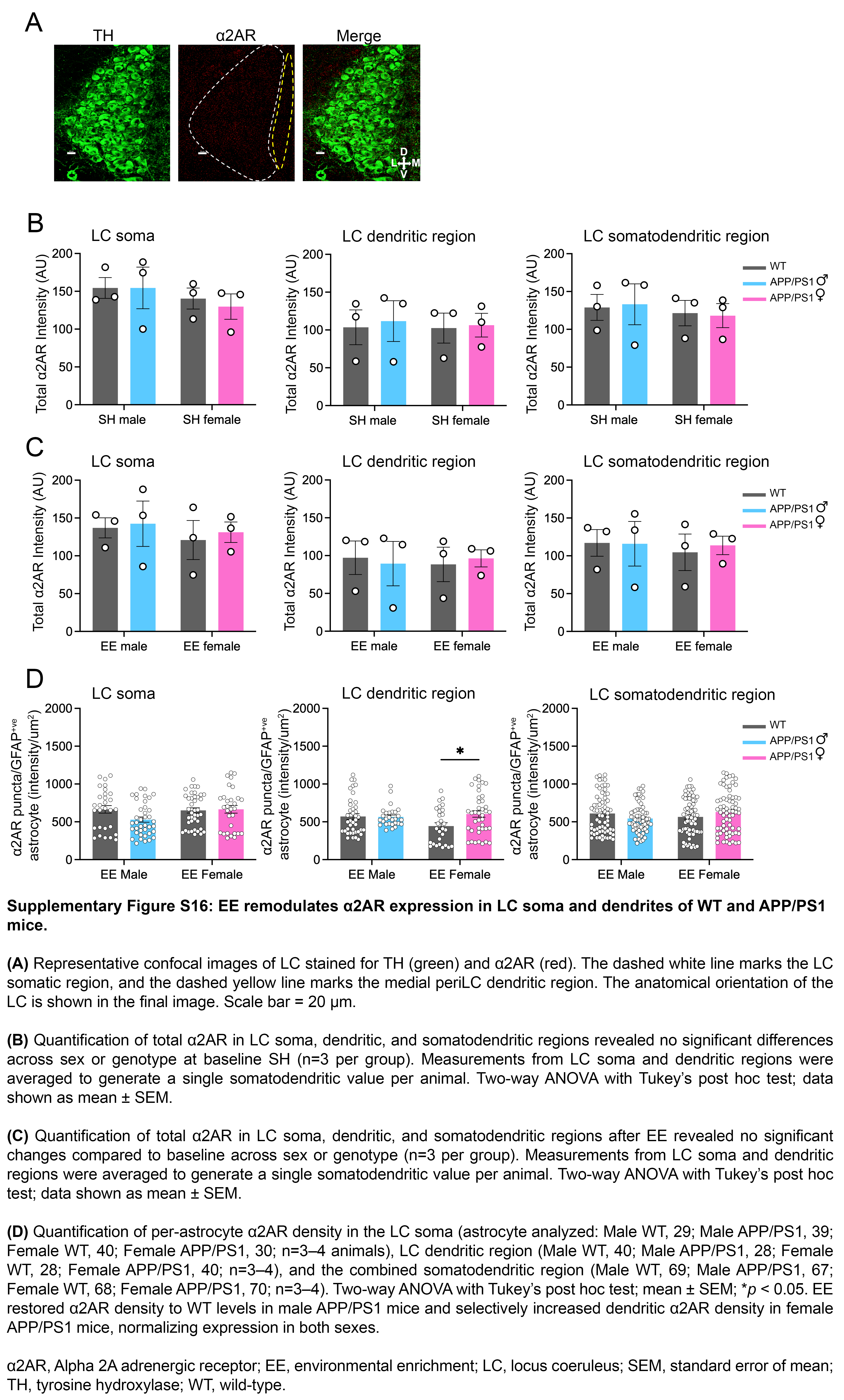

Supplement: Supplementary file 19 — Supporting Information [file ALZ-22-e71168-s003.tif]
